# Pilot investigations into the mechanistic basis for adverse effects of glucocorticoids in dysferlinopathy

**DOI:** 10.1186/s13395-024-00350-6

**Published:** 2024-08-09

**Authors:** Erin M. Lloyd, Rachael C. Crew, Vanessa R. Haynes, Robert B. White, Peter J. Mark, Connie Jackaman, John M. Papadimitriou, Gavin J. Pinniger, Robyn M. Murphy, Matthew J. Watt, Miranda D. Grounds

**Affiliations:** 1 Department of Anatomy, Physiology and Human Biology, School of Human Sciences, The University of Western Australia, Perth, WA Australia; 2 Curtin Health Innovation Research Institute, Curtin Medical School, Curtin University, Bentley, WA Australia; 3 Department of Obstetrics and Gynaecology, University of Cambridge, Cambridge, UK; 4 Department of Anatomy and Physiology, Faculty of Medicine Dentistry and Health Sciences, University of Melbourne, Parkville, VIC Australia; 5 MD Education Unit, UWA Medical School, The University of Western Australia, Perth, WA Australia; 6 Department of Pathology and Laboratory Medicine, UWA Medical School, The University of Western Australia, Perth, WA Australia; 7 Department of Biochemistry and Chemistry, School of Agriculture, Biomedicine and Environment, La Trobe University, Melbourne, VIC Australia

**Keywords:** Limb-girdle muscular dystrophy, Dysferlinopathy, Dysferlin, Glucocorticoids, Dexamethasone, Skeletal muscle, Adipocytes, Complement, Inflammasome, Glycogen

## Abstract

**Background:**

Dysferlinopathies are a clinically heterogeneous group of muscular dystrophies caused by gene mutations resulting in deficiency of the membrane-associated protein dysferlin. They manifest post-growth and are characterised by muscle wasting (primarily in the limb and limb-gridle muscles), inflammation, and replacement of myofibres with adipose tissue. The precise pathomechanism for dysferlinopathy is currently unclear; as such there are no treatments currently available. Glucocorticoids (GCs) are widely used to reduce inflammation and treat muscular dystrophies, but when administered to patients with dysferlinopathy, they have unexpected adverse effects, with accelerated loss of muscle strength.

**Methods:**

To investigate the mechanistic basis for the adverse effects of GCs in dysferlinopathy, the potent GC dexamethasone (Dex) was administered for 4–5 weeks (0.5–0.75 µg/mL in drinking water) to dysferlin-deficient BLA/J and normal wild-type (WT) male mice, sampled at 5 (Study 1) or 10 months (Study 2) of age. A wide range of analyses were conducted. Metabolism- and immune-related gene expression was assessed in psoas muscles at both ages and in quadriceps at 10 months of age. For the 10-month-old mice, quadriceps and psoas muscle histology was assessed. Additionally, we investigated the impact of Dex on the predominantly slow and fast-twitch soleus and extensor digitorum longus (EDL) muscles (respectively) in terms of contractile function, myofibre-type composition, and levels of proteins related to contractile function and metabolism, plus glycogen.

**Results:**

At both ages, many complement-related genes were highly expressed in BLA/J muscles, and WT mice were generally more responsive to Dex than BLA/J. The effects of Dex on BLA/J mice included (i) increased expression of inflammasome-related genes in muscles (at 5 months) and (ii) exacerbated histopathology of quadriceps and psoas muscles at 10 months. A novel observation was pronounced staining for glycogen in many myofibres of the damaged quadriceps muscles, with large pale vacuolated myofibres, suggesting possible myofibre death by oncosis.

**Conclusion:**

These pilot studies provide a new focus for further investigation into the adverse effects of GCs on dysferlinopathic muscles.

**Supplementary Information:**

The online version contains supplementary material available at 10.1186/s13395-024-00350-6.

## Background

Dysferlinopathies are a heterogeneous class of autosomal recessive muscular dystrophies that arise from mutations in the dysferlin (*Dysf*) gene, causing deficiency of the dysferlin protein [1, 2]. Dysferlin is a membrane-associated protein present in a range of cell types. It interacts with many other proteins and plays important roles in protein vesicle trafficking and fusion (reviewed by Lek et al. [3] and Bulankina et al. [4]). In skeletal and cardiac muscles, dysferlin is typically localised to the sarcolemma and transverse tubules. It is strongly associated with transverse tubule formation [5–7] and forms a complex with dihydropyridine receptor and ryanodine receptors (reviewed by Bulankina et al. [4]). Clinically, dysferlinopathies present as several different phenotypes [2], with the most widely studied being limb-girdle muscular dystrophy (LGMD) R2 dysferlin-related (previously known as LGMD2B [8]) and Miyoshi myopathy, with initial weakness in the proximal limb-girdle or distal limb muscles, respectively [9, 10]; although, these phenotypes overlap [11]. The pathology predominantly affects skeletal muscles and usually manifests once growth ceases, typically in young adults [9].

Dysferlinopathies are characterised by progressive skeletal muscle weakness and wasting, inflammation, accumulation of intramyocellular lipid droplets, and later pronounced adipocyte replacement of myofibres in humans and mice [9, 12–15]. In humans, magnetic resonance imaging (MRI) studies demonstrate that the slow-twitch soleus muscle is the earliest and most affected muscle with respect to adipocyte replacement [14]. In dysferlin-deficient mice, including the BLA/J strain, the histopathology with lipid droplets and adipocyte replacement is evident by about 10 months in the most affected quadriceps, psoas, and gluteus muscles in the limb-girdle region and increasingly pronounced by 18 months of age [12, 13, 15–17]. Histopathology in other muscles, such as gastrocnemius and tibialis anterior, is also evident by 24 months [18, 19]. In marked contrast, dysferlin-deficient soleus and extensor digitorum longus (EDL) muscles show minimal overt histopathology, even in older mice [19, 20].

These histopathological observations accord with proteomic analyses identifying alterations in muscle damage, repair, and remodelling pathways and infiltration of inflammatory/immune cells in quadriceps from human dysferlinopathy patients [21] and dysferlin-deficient BLA/J mice [22], and also gene expression analyses of human dysferlinopathy patients [23]. Moreover, muscle damage, shown by increased creatine kinase levels in the blood (a systemic measure of myofibre leakiness and damage), is significantly increased in humans with dysferlinopathy (~ 20–200 fold) [2], and in BLA/J mice aged 6, 9, and 12 months (~ 3–5 fold) [15].

Interestingly, there is only a low incidence (< 1% by area) of myofibre necrosis in the affected dysferlin-deficient muscles, even in mice up to 19 months of age [12]; instead, some form of proteolysis is suggested as the primary cause for the loss of dysferlin-deficient myofibres [13, 24]. While overt histopathology is not evident in dysferlin-deficient mouse muscles aged 3–5 months, some central myonuclei [25], increased protein thiol oxidation, and high levels of lipofuscin (a measure of cumulative oxidative damage) are present in ‘affected’ muscles at 3 months of age [12], with marked alterations in lipid metabolism and skeletal muscle lipidome in such young mice [26]. Thus 3–5 months is a good age to investigate intrinsic disturbances in dysferlin-deficient mouse muscles, before secondary complications of histopathology occur and intensify in vulnerable muscles (i.e., quadriceps and psoas) from about 10 months of age [20]. It is still unclear how dysferlin deficiency initially results in the severe pathology of only specific muscles [1, 4]; consequently, no effective treatments are currently available for dysferlinopathy patients.

Because dysferlinopathies have a pronounced inflammatory phenotype (reviewed in Tidball et al. [27]) and were initially considered to resemble inflammatory myopathies [28], some patients were treated with synthetic glucocorticoids (GCs). These GCs, such as prednisone and deflazacort, are widely used to treat many inflammatory and neuromuscular disorders, including myopathies, such as myositis and Duchenne muscular dystrophy (DMD), where they offer many benefits, as well as some adverse side effects [29–33]. In contrast, dysferlinopathy patients do not experience benefits from GC treatment; instead, they exhibit an acceleration in the decline in muscle strength and mobility [34, 35], supporting earlier observations [28] and discussed in Quattrocelli et al. [33]. Similar adverse outcomes were reported for dysferlin-deficient mice in two studies using daily prednisone or prednisolone [36, 37].

The GCs such as prednisolone, deflazacort, and dexamethasone (Dex) have many short- and long-term benefits but also adverse effects on signalling pathways related to metabolism and the inflammatory response, which are influenced by the delivery regimen (reviewed in Herbelet et al. [38], Quattrocelli et al. [33], and Grounds et al. [39]). Furthermore, it is widely recognised that GCs can cause muscle atrophy [40]. Given the complexity of the action of GCs, the mechanistic basis for the adverse response of dysferlinopathy to classic GC treatments is unknown, but such insight could help identify the molecular events driving this progressive dystropathology.

GCs are well-known regulators of lipid metabolism, promoting lipogenesis and adipogenesis and decreasing lipolysis. Aberrant GC signalling has been linked to metabolic diseases, including type 2 diabetes and obesity [33, 41, 42], with a more pronounced effect on adipocyte expansion reported in injured muscles [43]. Hence, we hypothesised that these molecular aspects might be rapidly exacerbated by GC treatment in dysferlinopathy and may manifest as the functional decline of dysferlin-deficient muscles within a short treatment period.

To investigate the adverse effects of short-term GCs on dysferlin-deficient muscles in vivo, Dex was given to 5 and 10-month-old BLA/J and wild-type (WT) male mice for 4 or 5 weeks. The impact of Dex treatment was assessed via measures of phenotype and gene expression (related to metabolism and immune system) in quadriceps and psoas muscles. Additionally, for the older mice (10 months), we also examined the impact of Dex on the histopathology of quadriceps and psoas muscles. Since loss of muscle strength is the key clinical indicator for adverse effects of GCs on patients with dysferlinopathy, Study 2 focused on muscle function. Detailed analyses were also conducted on the predominantly slow-twitch soleus and fast-twitch EDL muscles. In these muscles, we measured aspects of contractile function ex vivo, along with myofibre-type composition, and protein levels related to calcium (Ca^2+^) handling (in excitation-contraction coupling) and metabolism. Some novel observations emerged although Dex had little impact on these parameters.

## Methods

### Animals

Two in vivo studies were conducted using dysferlin-deficient BLA/J mice and normal male C57BL6/J WT mice: the background strain for BLA/J mice. Study 1 used young adult mice aged 4–5 months (*n* = 8 mice/group), and Study 2 used older adult mice aged 9–10 months where histopathology is more evident (*n* = 8–9 mice/group). Mice were obtained from the Animal Resources Centre (Murdoch, Western Australia) and housed at the Preclinical Animal Facility (University of Western Australia) under a 12-hour light-dark cycle at an ambient temperature of 20–22 °C, with free access to water and standard rodent chow. All mice were acclimatised for one week before the start of the experiment. The young mice (Study 1) were caged in groups of eight. However, the older mice (aged 10 months; Study 2) were housed individually, in adjacent cages with clear sides, due to aggression and fighting of the BLA/J mice. All animal procedures were approved by the Animal Ethics and Experimentation Committee of the University of Western Australia (RA/3/100/1436), in accordance with guidelines of the National Health and Medical Research Council of Australia.

### Dex treatment of mice

Mice were treated with the GC Dex as dexamethasone acetate (Sigma-Aldrich Chemical Co., St Louis, MO, USA) dissolved in drinking water [44]. Untreated mice received normal drinking water. Water was replaced twice weekly, and water consumption was recorded throughout treatment.

Dex is widely used clinically and in animal studies [45], and is approximately 50-fold more potent at activating the GC receptor than corticosterone (the major adrenal corticosteroid in mice) [46]. Dex delivery in drinking water is far less stressful for mice than acute administration by daily oral gavage or intraperitoneal injection during the daytime (widely used in animal studies). Moreover, consumption of Dex in water accords with the onset of normal nocturnal activity (during the dark phase) with drinking and circadian influences on metabolism in the mice: it is noted that the adrenal cortex produces endogenous corticosterone in response to circadian and stress stimuli [47].

Many rodent studies with Dex use short-term administration, for example, 8–10 days [44]. In contrast, we aimed to treat mice for several weeks to better reflect chronic treatment regimens and maximise the opportunity for alterations in measures used in these studies. Since Dex (like other GCs) is known to cause muscle atrophy [40, 48, 49], there was concern that muscle wasting and consequent loss of body mass might be more pronounced in dysferlin-deficient mice, with implications for the continuation of the experiment due to ethical restrictions (i.e., experiment termination at > 15% body mass loss).

In Study 1, young adult WT and BLA/J mice were untreated or given 0.5 µg/mL Dex in drinking water for 5 weeks, beginning at 4 months with sacrifice at 5 months of age. There was no pronounced loss of body mass in this pilot study. Thus, a slightly higher dose of Dex was used in the larger Study 2 to assess the impact more thoroughly at a more advanced stage of the disease, albeit over a shorter time. Older mice were untreated or given 0.75 µg/mL Dex in water for 4 weeks, beginning at 9 months with sacrifice at 10 months of age.

### Tissue sampling

Following Dex treatment, mice were anesthetised via intraperitoneal injection of sodium pentobarbitone (40 mg/kg of body mass (BM)) and weighed. For Study 1, quadriceps and psoas muscles were dissected, weighed, and, in most cases, immediately snap frozen in liquid nitrogen and stored at -80 °C until processed for molecular analyses.

For Study 2 (aged 10 months), the soleus and EDL muscles (from one leg) were dissected under anaesthesia and used for functional analyses (described below), with the contralateral muscles snap-frozen for subsequent protein analyses. In addition, quadriceps and psoas muscles were excised (one of each muscle per mouse for all BLA/J mice and untreated WT mice) and fixed in 4% paraformaldehyde for standard processing into paraffin blocks for histological analyses. Animals were euthanised via an overdose of intraperitoneal sodium pentobarbitone (> 160 mg/kgBM).

### Gene expression in quadriceps and psoas muscles (Studies 1 and 2)

Expression of selected genes associated with metabolism, adipogenesis, inflammation, and the complement pathway (see Supplementary Table S1) was measured by reverse transcription quantitative polymerase chain reaction (RT-qPCR). Genes of interest were selected related to metabolism, with a focus on lipogenesis and adipogenesis, and also genes associated with the immune system since increased inflammation and activation of the complement system are widely reported in dysferlinopathy [50–52].

The gene expression analyses for Study 1 were conducted at the University of Melbourne and used a different methodology from Study 2, which was conducted at the University of Western Australia. Consequently, it is not appropriate to directly compare expression levels between the two studies. For both studies, total RNA was extracted from muscle tissues using the Qiazol method, per the manufacturer’s instructions (Qiagen, VIC, Australia). The concentration and purity of RNA were assessed using the Nanodrop ND-1000 spectrophotometer (Thermo Scientific, Wilmington, DE, USA).

Study 1 assessed gene expression in psoas muscle from 5-month-old mice. Total RNA (1 µg) was converted to cDNA using an iSCRIPT kit (Bio-Rad, Hercules, CA, USA). Each 10 µl RT-qPCR reaction consisted of 5 µl SYBR Green Master Mix (Qiagen, Hilden, Germany), 1 µl forward and reverse gene-specific primers (20 µM; either Qiagen or Sigma-Aldrich; see Supplementary Table S1), 3 µl H_2_O, and 1 µl cDNA. The PCR reaction cycle included an incubation period of 10 min at 95 °C, denaturation for 5 s at 95 °C, annealing for 20 s at 60 °C, and elongation for 20 s at 72 °C; this was performed using a CFX384 Touch Real-Time PCR Detection System (Biorad). Skeletal actin (*Acta1*) was used as a reference gene to normalise gene expression values in the psoas muscle, and hypoxanthine-guanine phosphoribosyltransferase (*Hprt*) was used as a reference gene for the liver. Relative gene expression was calculated using the 2^−ΔΔCT^ Method, and values were normalised to the untreated WT for each gene [53].

Study 2 assessed gene expression in the quadriceps and psoas muscles of 10-month-old mice using a Rotorgene Q machine (Qiagen). Total RNA (1 µg) was converted to cDNA using a QuantiTect Reverse Transcription Kit (Qiagen). All gene-specific primer sets were supplied by Qiagen (QuantiTect Primer Assay, Qiagen; see Supplementary Table S1). Each 10 µl PCR reaction consisted of 2 µl QuantiTect primer, 5 µl Qiagen SYBR green pre-mix, 2 µl ddH_2_O, and 1 µl cDNA template. The two-step PCR reaction cycle included an incubation period of 5 min at 95 °C, denaturation for 15 s at 95 °C, and combined annealing and elongation steps for 30 s at 60 °C. Standard curves were generated from 10-fold serial dilutions of PCR product and were used to determine relative gene expression concentration with Rotorgene Q series software. All mRNA expression values were standardised against the reference genes peptidylprolyl isomerase A (*Ppia*) and TATA-box binding protein (*Tbp1*) using the GeNorm algorithm [54].

### Muscle function assessment for older mice (10 months; Study 2)

#### Grip strength

Forelimb grip strength was measured in the 10-month-old mice using a Chatillon Digital Force Gauge (Model DFE-002; AMETEK, FL, USA) as previously described [55]. The mean of four consecutive grip strength tests was recorded and normalised to body mass (g/gBM). Measures of grip strength and body mass were made weekly throughout the treatment period and on the morning of sampling for all experimental groups.

#### Contractile function of ex vivo soleus and EDL muscles

Due to the marked differences in contractile and metabolic properties of slow- and fast-twitch myofibres, and since most muscles are a complex mixture of different myofibre types [56, 57], we compared the predominantly slow soleus muscle composed of about 37% slow (type 1) and 63% intermediate (type 2A/2X) myofibres, with the predominantly fast EDL muscle composed of about 34% intermediate and 66% fast (type 2B) myofibres [58]. Notably, the mouse soleus muscle most closely resembles the relatively slow human muscles [59].

Ex vivo soleus and EDL contractile function analyses were conducted as described previously [55]. Briefly, the excised muscles were mounted in an in vitro force transducer system (1205 A, Aurora Scientific Inc., Aurora, Canada) in an organ bath filled with mammalian Ringer solution, bubbled with Carbogen (95% O_2_, 5% CO_2_), and maintained at 25 °C. Contractile function measures included peak isometric twitch force, time to peak twitch force, half-relaxation time, maximum rate of force production, the force-frequency relationship including maximum tetanic force, and muscle fatigability and post-fatigue recovery. Force (N) was normalised to cross-sectional area, calculated from myofibre length, muscle mass, and density (1.056 g/cm^3^), and presented as specific force (N/cm^2^).

### Molecular analyses of soleus and EDL muscle from older mice (10 months; Study 2)

#### Myofibre typing: MyHC composition

Myosin heavy chain (MyHC) composition of 10-month soleus and EDL muscles was determined by quantifying proteins for four distinct MyHC isoforms, ranging from slow to fast as MyHC 1, 2 A, 2X, and 2B, in muscle homogenates as described previously [55]. Briefly, the muscle homogenates were denatured, and 20 µg total protein was separated using SDS-PAGE, adapted for optimal separation of the four MyHC isoforms, as described previously [55, 60]. Gels were stained with Coomassie brilliant blue G250 to visualise the MyHC protein bands, images were collected, and densitometry of each band performed using the Chemidoc MP system and Image Lab version 6.0 (Bio-Rad).

#### Quantification of protein abundance and glycogen content

In the same soleus and EDL muscles as above, immunoblotting was used to quantify the abundance of various proteins related to Ca^2+^ handling and metabolism, as described previously [60–62], with western blot signals of given proteins normalised to total protein [63]. Additionally, the glycogen content of these muscles was assessed biochemically, as described in detail previously [60, 61]. Protein and glycogen data for the untreated WT and BLA/J soleus and EDL muscles, including marked changes in protein levels, have recently been described [61].

### Histology for quadriceps and psoas muscles from older mice (10 months; Study 2)

Histopathology was assessed using sections of fixed paraffin blocks of quadriceps and psoas muscles from 10-month-old WT (*n* = 9), untreated BLA/J (*n* = 7), and Dex-treated BLA/J mice (BLA/J + Dex; *n* = 8). Note that muscles were unavailable for Dex-treated WT mice (WT + Dex). Transverse sections were cut (7 μm thick) and stained with haematoxylin & eosin (H&E) for standard descriptive histology. Due to high variability within sections of individual BLA/J muscles (see Results), it was not possible to formally quantify the extent of histopathology across all muscles.

Staining for glycogen was also conducted using classic periodic acid-Schiff (PAS) stain in combination with diastase (PAS-D) to digest the glycogen and confirm the specificity of the staining. Normal WT mouse liver was used as a positive control for glycogen (PAS and PAS-D staining). In addition, for comparison with another form of muscular dystrophy, sections from quadriceps muscle of the *mdx* mouse model for Duchenne muscular dystrophy were stained for glycogen.

Images were captured using a Nikon Eclipse Ti inverter microscope equipped with Nikon DS-Fi2 camera (Nikon Corporation, Japan) and NIS-Elements Advanced Research 3.0 Software (Nikon Instruments Inc., Japan).

### Statistical analysis

All data are presented as mean ± standard deviation (SD). Gene expression data were log_10_ transformed, where necessary, to improve normality. The impact of Dex treatment on dysferlin deficiency was examined using separate three-way and two-way Analyses of Variance (ANOVA) or unpaired two-tailed t-tests where appropriate. *Post hoc* comparisons with Holm-Bonferroni corrections were conducted for each statistical test, where appropriate, with statistical significance taken as *p* ≤ 0.05. Statistical analyses were performed using Jamovi (Version 1.6).

## Results

### Body and tissue mass

#### Young adult mice (5 months; Study 1)

**Fig. 1:**
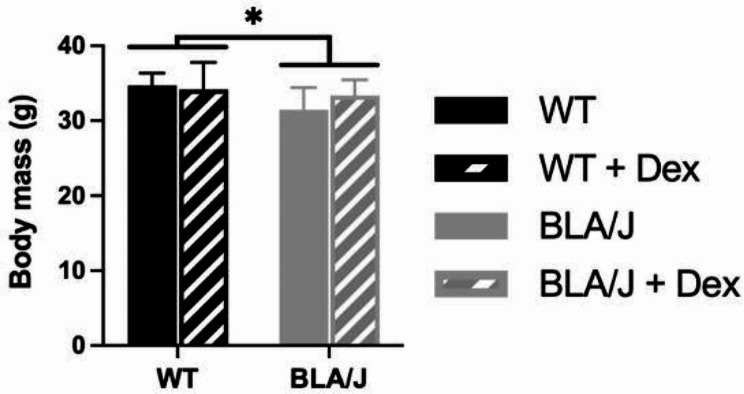
Whole body mass of wild-type (WT) and dysferlin-deficient BLA/J mice aged 5 months, without and with dexamethasone (Dex) treatment. Data were analysed by two-way ANOVA: * BLA/J (± Dex) vs. WT (± Dex) (*p* < 0.05, strain effect). Data are presented as mean ± SD (*n* = 8)

Following 5 weeks of no treatment or Dex treatment (0.5 µg/mL), 5-month-old BLA/J mice weighed less than WT overall (*p* < 0.05; Fig. 1).

Food and water consumption throughout this experiment was higher in the groups of untreated BLA/J than untreated WT mice (*n* = 8 mice/group), while Dex treatment reduced food and water consumption in the groups of BLA/J mice but not in WT (*p* < 0.0001; Supplementary Fig. S1).

### Older adult mice (10 months; Study 2)

**Fig. 2:**
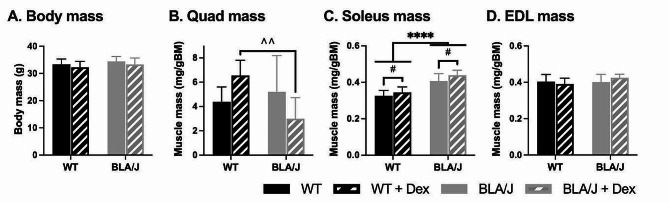
Body and muscle mass of WT and dysferlin-deficient BLA/J mice aged 10 months, without and with dexamethasone (Dex) treatment. **A** Body mass measured at sampling, and **B** quadriceps (quad), **C** soleus, and **D** extensor digitorum longus (EDL) muscle mass normalised to body mass (mg/gBM). Data were analysed by two-way ANOVA: **** BLA/J (± Dex) vs WT (± Dex) (*p* < 0.0001, strain effect); # Dex-treated vs. untreated (*p* < 0.05, treatment effect); ^^ BLA/J + Dex vs WT + Dex (*p* < 0.01, strain/treatment interaction effect). Data are presented as mean ± SD (*n* = 8–9)

Following 4 weeks of no treatment or Dex treatment (0.75 µg/mL), body mass of the 10-month-old WT and BLA/J mice (both untreated and treated) did not differ (Fig. 2A). Quadriceps muscle mass did not differ between untreated mice but was significantly lower (~ -55%) in Dex-treated BLA/J mice compared with Dex-treated WT (*p* < 0.05; Fig. 2B). Soleus mass was significantly heavier in BLA/J mice compared to WT overall (*p* < 0.0001) and also in Dex-treated compared to untreated mice overall (*p* < 0.05; Fig. 2C). EDL mass was no different between groups (Fig. 2D).

### Metabolism-associated gene expression

#### Young adult mice (5 months)

**Fig. 3:**
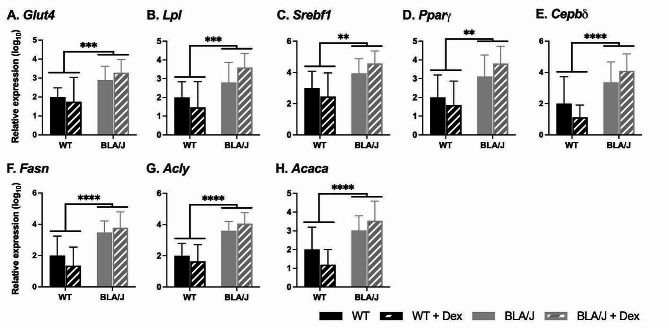
Relative metabolism-associated gene expression in psoas muscle of wild-type (WT) C57Bl/6J and dysferlin-deficient BLA/J mice aged 5 months, without and with dexamethasone (Dex) treatment. **A** Glucose transporter type 4 (*Glut4*); **B** Lipoprotein lipase (*Lpl*); **C** Sterol regulatory element-binding transcription factor 1 (*Srebf1*); **D** Peroxisome proliferator-activated receptor gamma (*Pparγ*); **E** CCAAT/enhancer-binding protein delta (*Cepbδ*); **F** Fatty Acid Synthase (*Fasn*); **G** ATP citrate lyase (*Acly*); **H** Acetyl-CoA carboxylase 1 (*Acaca*). Skeletal actin (*Acta1*) was used as a reference gene to normalise gene expression values in the psoas muscle. Relative gene expression was calculated using the 2^−ΔΔCT^ Method, and values were normalised to the untreated WT for each gene. Data were analysed by two-way ANOVA: **, ***, **** BLA/J (± Dex) vs WT (± Dex) (*p* < 0.01, 0.001, 0.0001, respectively, strain effect). Data are log_10_ transformed and presented on a log_10_ *y*-axis scale as mean ± SD (*n* = 5–8)

For 5-month-old mice, the impact of Dex treatment on metabolism-associated gene expression was assessed in the psoas muscle (Fig. 3). BLA/J mice had higher expression of *Glut4*, *Lpl*, *Srebf1*, *Pparγ*, *Cepbδ*, *Fasn*, *Acly*, and *Acaca* compared to WT mice overall (*p*s < 0.05; Fig. 3). However, Dex treatment did not significantly affect the expression of those genes in either mouse strain.

#### Older adult mice (10 months)

**Fig. 4:**
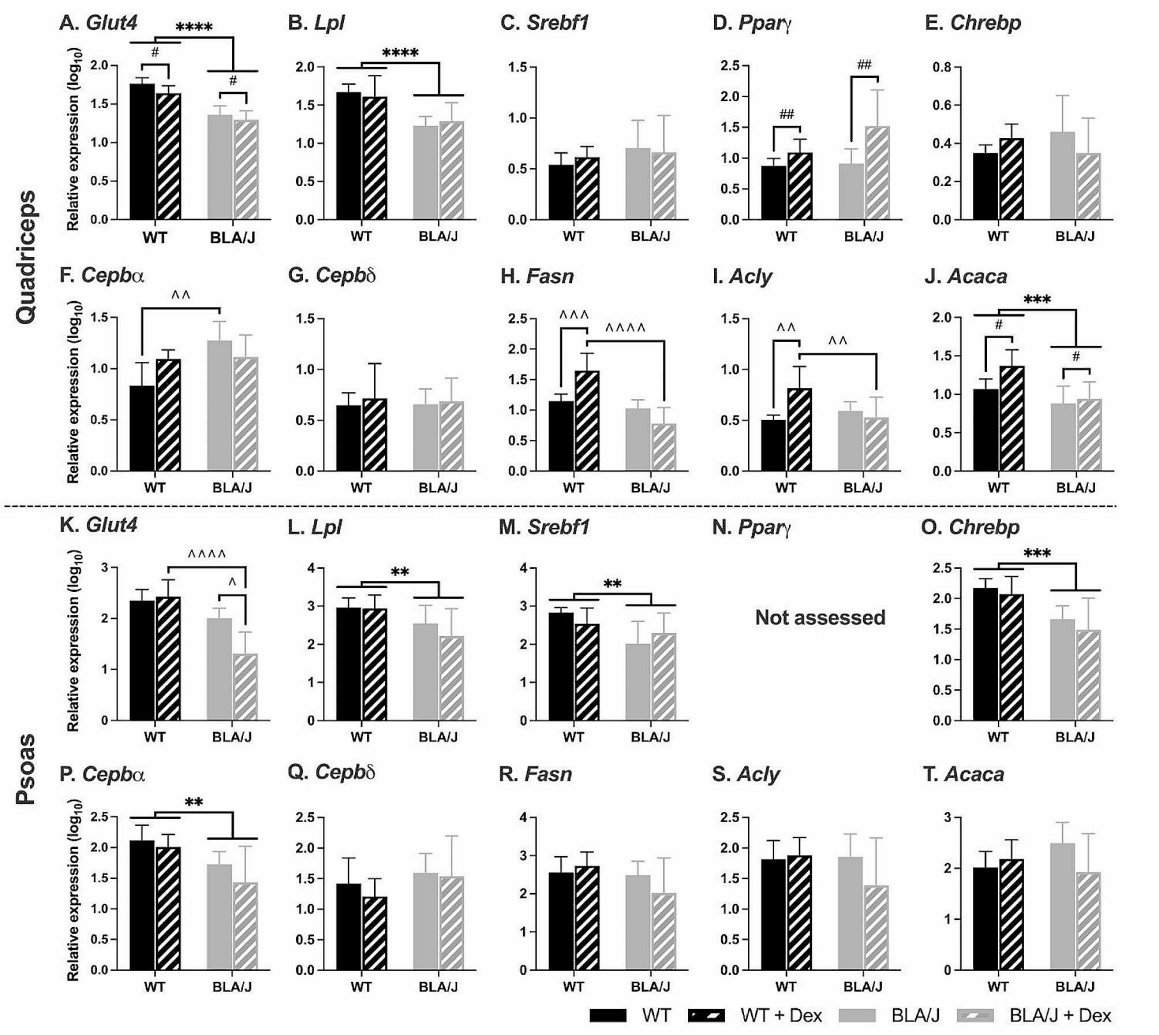
Relative metabolism-associated gene expression in quadriceps and psoas muscles of wild-type (WT) C57Bl/6J and dysferlin-deficient BLA/J mice aged 10 months, without and with dexamethasone (Dex) treatment. **A**, **K** Glucose transporter type 4 (*Glut4*); **B**, **L** Lipoprotein lipase (*Lpl*); **C**, **M** Sterol regulatory element-binding transcription factor 1 (*Srebf1*); **D** Peroxisome proliferator-activated receptor gamma (*Pparγ*); **E**, **O** Carbohydrate-responsive element-binding protein (*Chrebp*); **F**, **P** CCAAT enhancer binding protein alpha (*Cebpα*); **G**, **Q** CCAAT/enhancer-binding protein delta (*Cepbδ*); **H**, **R** Fatty Acid Synthase (*Fasn*); **I**, **S** ATP citrate lyase (*Acly*); **J**, **T** Acetyl-CoA carboxylase 1 (*Acaca*). All mRNA expression values were standardised against the reference genes peptidylprolyl isomerase A (*Ppia*) and TATA-box binding protein (*Tbp1*) using the GeNorm algorithm [64]. Data were analysed by two-way ANOVA: **, ***, **** BLA/J (± Dex) vs WT (± Dex) (*p* < 0.01, 0.001, 0.0001, respectively, strain effect); #, ## Dex-treated vs untreated (*p* < 0.05, 0.01, respectively, treatment effect); ^, ^^, ^^^, ^^^^ significant difference between groups (*p* < 0.05, 0.01, 0.001, 0.0001, respectively, strain/treatment interaction effect). Data are log_10_ transformed and presented on a log_10_ *y*-axis scale as mean ± SD (*n* = 7)

For 10-month-old mice, the impact of Dex treatment on metabolism-associated gene expression was assessed in the quadriceps and psoas muscles (Fig. 4).

BLA/J quadriceps had lower *Glut4*,* Lpl*, and *Acaca* expression compared with WT overall (*p*s < 0.05; Fig. 4A, B, J), and higher *Cebpα* expression in untreated BLA/J than in WT (Fig. 4F). Dex-treated quadriceps had lower *Glut4* expression and higher *Pparγ* and *Acaca* expression than untreated (for both strains; *p* < 0.05; Fig. 4A, D, J). Dex had WT-specific effects where Dex-treated WT quadriceps had higher *Fasn* and *Acly* expression compared with untreated WT quadriceps muscles (*p*s < 0.05; Fig. 4H, I), but Dex did not have strain-specific effects on BLA/J quadriceps muscles. *Srebf1*, *Chrebp*, and *Cepbδ* expression in the quadriceps were unaffected by strain or Dex treatment.

BLA/J psoas muscles had lower *Lpl*, *Srebf1*, *Chrebp*, and *Cebpα* expression than WT overall (*p*s < 0.05; Fig. 4L, M, O, P). Dex had a strain-specific impact on *Glut4* expression, which was lower in Dex-treated BLA/J muscles than in untreated BLA/J and Dex-treated WT (*p* < 0.05; Fig. 4K). *Cepbδ*, *Fasn*, *Acly*, and *Acaca* expression in the psoas were not altered.

### Immune-associated gene expression

#### Young adult mice (5 months)

**Fig. 5:**
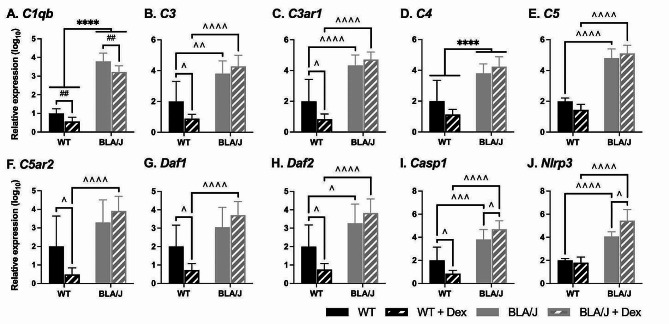
Relative immune-associated gene expression in psoas muscle of wild-type (WT) C57Bl/6J and dysferlin-deficient BLA/J mice aged 5 months, without and with dexamethasone (Dex) treatment. **A** Complement C1q B Chain (*C1qb*); **B** Complement component 3 (*C3*); **C** Complement C3a Receptor 1 (*C3ar1*); **D** Complement component 4 (*C4*); **E** Complement component 5 (C5); **F** Complement C5a Receptor 2 (*C5ar2*); **G** Decay-accelerating factor 1 for complement (*Daf1* or CD55); **H** Decay-accelerating factor 2 for complement B (*Daf2* or CD55b); **I** Caspase 1 (*Casp1*); **J** NLR family pyrin domain containing 3 (*Nlrp3*). Skeletal actin (*Acta1*) was used as a reference gene to normalise gene expression values in the psoas muscle. Relative gene expression was calculated using the 2^−ΔΔCT^ Method, and values were normalised to the untreated WT for each gene. Data were analysed by two-way ANOVA: **** BLA/J (± Dex) vs WT (± Dex) (*p* < 0.0001, strain effect); ## Dex-treated vs untreated (*p* < 0.01, treatment effect); ^, ^^, ^^^, ^^^^ significant difference between groups (*p* < 0.05, 0.01, 0.001, 0.0001, respectively, strain/treatment interaction effect). Data are log_10_ transformed and presented on a log_10_ *y*-axis scale as mean ± SD (*n* = 5–8)

The impact of Dex treatment on immune-associated gene expression in the psoas muscle of 5-month-old mice was assessed (Fig. 5). Overall, there were several marked strain differences: for untreated mice with generally much higher gene expression in the BLA/J compared with WT psoas. Dex significantly reduced the expression of most genes in WT psoas, but such reduction was not evident in BLA/J psoas (apart from *C1qb*).

BLA/J mice had higher *C1qb* and *C4* expression than WT overall (*p*s < 0.05; Fig. 5A, D). Dex-treated psoas had lower expression of *C1qb* compared with untreated (for both strains; *p* < 0.05; Fig. 5A). Both untreated and Dex-treated BLA/J psoas muscles had higher *C3*, *C3ar1*, *C5*, *Daf2*, *Casp1*, and *Nlrp3* compared to WT mice (*p*s < 0.05; Fig. 5B, C, E, H-J). Furthermore, Dex-treated BLA/J had higher expression of *C5ar2* and *Daf1* than Dex-treated WT (*p*s < 0.05; Fig. 5F, G). Additionally, Dex had WT-specific effects, with lower expression of the complement-related genes *C3*, *C3ar1*, *C5ar2*, *Daf1*, *Daf2*, and *Casp1* in Dex-treated compared with untreated WT mice (*p*s < 0.05; Fig. 5B, C, F-I). Dex also had a BLA/J-specific effect on the expression of the inflammasome-associated genes *Casp1* and *Nlrp3*, which was higher in Dex-treated than in untreated BLA/J mice (*p*s < 0.05; Fig. 5I, J).

#### Older adult mice (10 months)

**Fig. 6:**
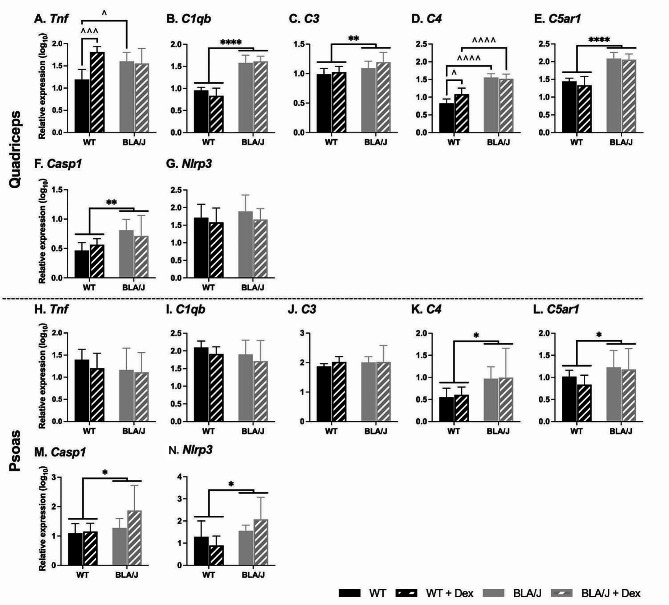
Relative immune-related gene expression in quadriceps and psoas muscles of wild-type (WT) C57Bl/6J and dysferlin-deficient BLA/J mice aged 10 months, without and with dexamethasone (Dex) treatment. **A**, **H** Tumour necrosis factor (*Tnf*); **B**, **I** Complement C1q B Chain (*C1qb*); **C**, **J** Complement component 3 (*C3*); **D**, **K** Complement component 4 (*C4*); **E**, **L** Complement C5a Receptor 1 (*C5ar1*); **F**, **M** Caspase 1 (*Casp1*); **G**, **N** NLR family pyrin domain containing 3 (*Nlrp3*). All mRNA expression values were standardised against the reference genes peptidylprolyl isomerase A (*Ppia*) and TATA-box binding protein (*Tbp1*) using the GeNorm algorithm [64]. Data were analysed by two-way ANOVA: *, **, **** BLA/J (± Dex) vs WT (± Dex) (*p* < 0.05, 0.01, 0.0001, respectively, strain effect); # Dex-treated vs untreated (*p* < 0.05, treatment effect); ^, ^^^, ^^^^ significant difference between groups (*p* < 0.05, 0.001, 0.0001, respectively, strain/treatment interaction effect). Data are log_10_ transformed and presented on a log_10_ *y*-axis scale as mean ± SD (*n* = 4–10)

For 10-month-old mice, the impact of Dex treatment on immune-associated gene expression was assessed in the quadriceps and psoas muscles (Fig. 6).

BLA/J quadriceps had higher *C1qb*, *C3*, *C5ar1*, and *Casp1* expression than WT overall (*p*s < 0.05; Fig. 6B, C, E, F). Furthermore, for untreated BLA/J mice, *Tnf* and *C4* expression was higher than in untreated WT, and C4 expression was higher in Dex-treated BLA/J than in Dex-treated WT (*p*s < 0.05; Fig. 6A, D). Dex treatment had a strain-specific effect in the WT quadriceps, where *C4* expression was higher in Dex-treated compared with untreated WT (*p* < 0.05; Fig. 6D). *Nlrp3* expression in the quadriceps was not altered.

BLA/J psoas muscles had higher *C4*, *C5ar1*, *Casp1*, and *Nlrp3* expression than WT overall (*p*s < 0.05; Fig. 6K-M). *Tnf*, *C1qb*, and *C3* expression in the psoas were unaffected by dysferlin deficiency, with Dex having no effect on the expression of any genes.

We also examined the impact of Dex treatment on the gene expression of some metabolism (Supplementary Fig. S2, Supplementary Fig. S3) and immune-associated genes (Supplementary Fig. S4, Supplementary Fig. S5) for livers from the 5- and 10-month-old mice. There were limited effects of Dex on liver gene expression, and for reasons of space, these data are not discussed further here.

### Histopathology of quadriceps and psoas muscles from older adult WT mice (10 months)

#### Histopathology

**Fig. 7:**
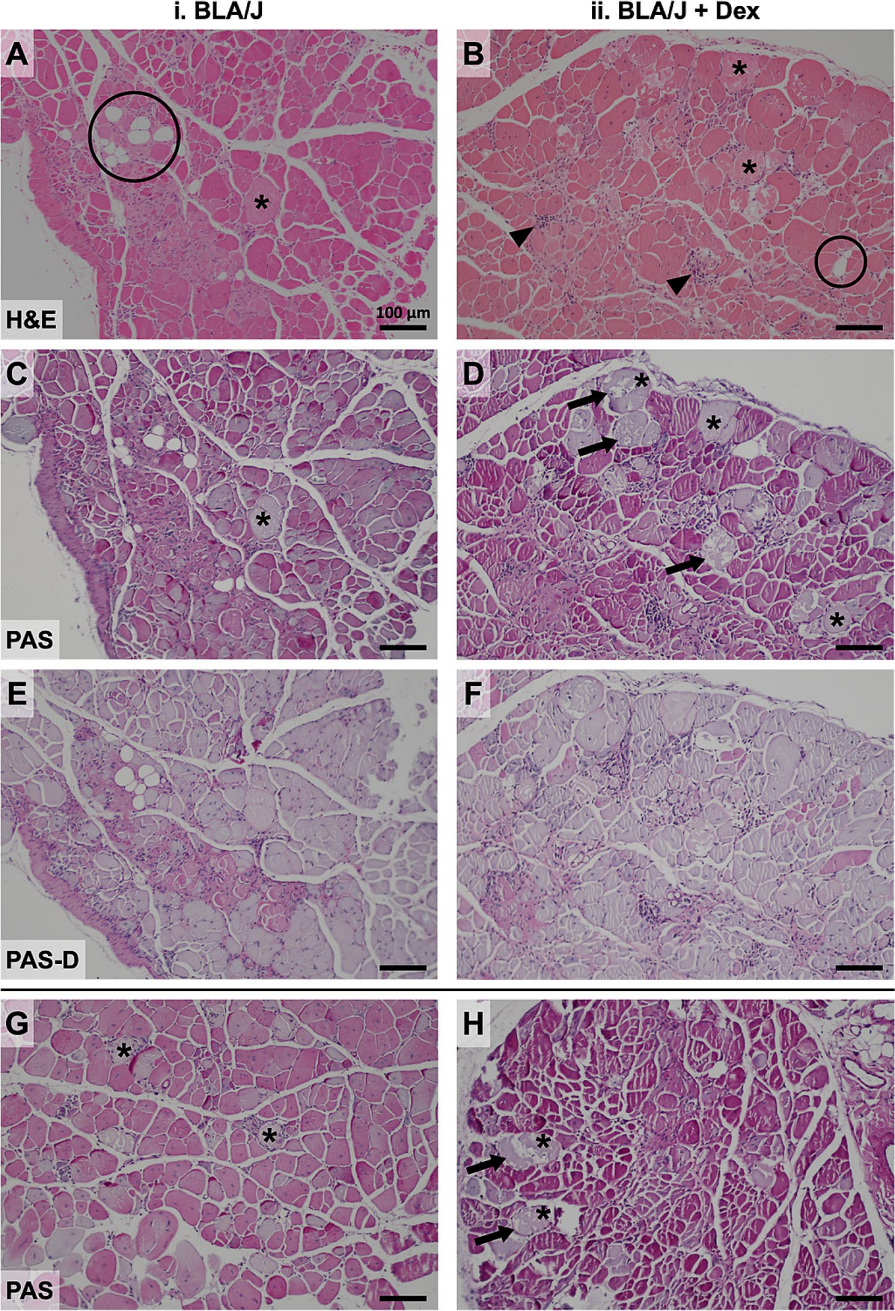
Effect of dexamethasone (Dex) treatment on histopathology of quadriceps muscles of BLA/J mice (aged 10 months). Representative images of consecutive paraffin sections showing highly variable histopathology of (i) BLA/J and (ii) BLA/J + Dex quadriceps stained by (**A**, **B**) haematoxylin and eosin (H&E), indicating the location of some adipocytes (circled), foci of inflammatory cells (arrowheads), and large pale myofibres (asterisks, *). **C**, **D** The presence of glycogen is shown by periodic acid-Schiff (PAS) staining, with (**E**, **F**) the absence of glycogen following diastase-induced breakdown (as a control to verify glycogen PAS staining; PAS-D). More (**G**, **H**) PAS-stained sections are shown from additional BLA/J mice. Dex treatment (in **B**, **D**, and **H**) appeared to increase the number of large pale myofibres (some marked by *), and some with conspicuous vacuoles/fragmentation (arrows) evident with PAS staining. In contrast, such pale myofibres did not appear as large (nor vacuolated) in untreated BLA/J muscle. Shown for four 10-month-old male mice: (i) BLA/J (ID for panels A, C, E. 16/21, and G. 16/23) and (ii) BLA/J + Dex (ID for panels B, D, F. 16/32, and H. 16/31). Scale bar = 100 μm

The untreated 10-month-old BLA/J quadriceps muscles had marked evidence of histopathology, with high variability within a single section, evident with both H&E and PAS staining (Fig. 7, Supplementary Fig. S6A-F). Pronounced histopathology was also seen for the BLA/J psoas muscles (Supplementary Fig. S6G-L, Supplementary Fig. S7). The histopathological features (as illustrated in Fig. 7) included variable myofibre sizes with occasional groups of small myofibres and large pale myofibres. There were also inflammatory cells (scattered and foci), often in interstitial tissue, and adipocytes of different sizes throughout the muscle tissue. In some cases, adipose tissue was conspicuous at the edge of the muscle tissue.

Although we did not expect the short duration of Dex treatment (only 4 weeks) to result in pronounced morphological changes in the BLA/J muscles, Dex appeared to exacerbate the pathology for quadriceps (as shown with images from Dex-treated BLA/J mice in Fig. 7). For the BLA/J + Dex psoas muscles (Supplementary Fig. S7), some histopathological changes were similar, but there was often great heterogeneity with limited amounts of tissue from these small psoas muscles. The Dex-treated BLA/J muscles exhibited paler myofibres that were often larger, with evidence of vacuoles (or fragmented sarcoplasm); these myofibres were often not associated with inflammatory cells. Areas of large pale myofibres with sarcoplasmic disorder were pronounced at the edge of several quadriceps (e.g., Fig. 7D) and psoas (not shown) muscle sections, and there was more evidence of scattered adipocytes along the interstitial connective tissue, with several instances of quite large areas of adipose tissue at the edge (especially in the psoas) and generally more inflammatory cells.

#### Presence of glycogen

The large pale myofibres in BLA/J muscles (sometimes with vacuoles/fragmented) appeared ‘swollen’, indicating possible fluid uptake by the individual myofibre. To examine whether this ‘swelling’ might be due to increased glycogen within the myofibre [65], we stained serial paraffin sections for glycogen with PAS and PAS-D. The results showed a high intensity of glycogen staining in many myofibres for untreated BLA/J quadriceps and psoas muscles, which was not evident in WT muscles (Supplementary Fig. S6). Glycogen was not evident in the large pale myofibres, which were more conspicuous after Dex treatment and sometimes appeared more frequently at the edges of the muscles (Fig. 7D, Supplementary Fig. S7). This PAS staining exhibited more pronounced histopathological differences, especially after Dex treatment, and appeared to be more informative than classic H&E staining. The remnant staining seen in PAS-D (where glycogen has been removed by diastase digestion) probably represents high levels of glycoproteins/glycolipids, including mucins [66]: this was sometimes quite pronounced around thickened blood vessels and within muscles of BLA/J mice.

To further confirm the histopathology observed by PAS staining in BLA/J quadriceps and psoas muscles, we examined quadriceps muscles from *mdx* mice aged 2 months, where dystropathology is pronounced with many regenerated myofibres (with central myonuclei) and areas of active focal necrosis are often observed [25]; representing a distinctly different basis for dystropathology compared with dysferlinopathy (Supplementary Fig. S8). There was no striking glycogen staining in these *mdx* muscles, emphasising that high glycogen in dysferlin-deficient muscles (with marked histopathology) may be specific for this muscular dystrophy.

### Soleus and EDL muscles from older adult mice (10 months): function, MyHC composition, protein abundance, glycogen, and histology

#### Muscle function

To evaluate the effects of myofibre type on dysferlin deficiency and the response to Dex treatment, we examined the predominantly slow-twitch soleus and fast-twitch EDL muscles from the 10-month-old mice. The functional data for these muscles from untreated BLA/J and WT muscles are published [55]. Dex had limited effects on muscle function, which were mostly apparent in WT mice, with no effects specific to BLA/J muscles (Supplementary Fig. S9, Supplementary Fig. S10, Supplementary Fig. S11). Dex-treated soleus muscles (regardless of strain) had an increased rate of force production, while Dex-treated EDL muscles had a slower time to peak twitch force (Supplementary Fig. S10). In WT mice, Dex increased in vivo grip strength (Supplementary Fig. S9), and ex vivo, increased soleus tetanic force and reduced twitch contraction, relaxation time (Supplementary Fig. S10), and post-fatigue recovery (Supplementary Fig. S11). In WT EDL, Dex increased twitch relaxation time (Supplementary Fig. S10) and post-fatigue recovery and reduced the extent of fatigue force loss (Supplementary Fig. S11).

#### MyHC composition and Ca^2+^ handling- and metabolism-associated protein abundance

Since muscle function and metabolic properties are influenced by myofibre-type composition, we compared the effects of Dex exposure on the proportion of MyHC isoforms in the contralateral soleus and EDL muscles from mice reported above. Data for untreated mice were reported previously [55], and Dex treatment had no strain-specific effects (Supplementary Fig. S12). Soleus MyHC 2X composition was higher in BLA/J compared with WT muscles (regardless of treatment status; *p* < 0.05), and also higher in Dex-treated compared with untreated muscles (regardless of strain; *p* < 0.05). Similarly, Dex-treated EDL muscles, compared with untreated (regardless of strain), had a higher proportion of MyHC 2X and concurrently a lower proportion of MyHC 2B (*p*s < 0.001).

In the same muscles as above, we also assessed the impact of Dex treatment on the abundance of selected Ca^2+^-handling and metabolism-associated proteins. While there were marked effects of dysferlin deficiency on these proteins in untreated mice, as described previously [61], Dex treatment had little impact on the levels of these proteins (Supplementary Table S2). These data for Dex-treated mice are mentioned to demonstrate the scope of the molecular analyses conducted but do not warrant further discussion.

#### Muscle glycogen biochemical quantification

**Fig. 8:**
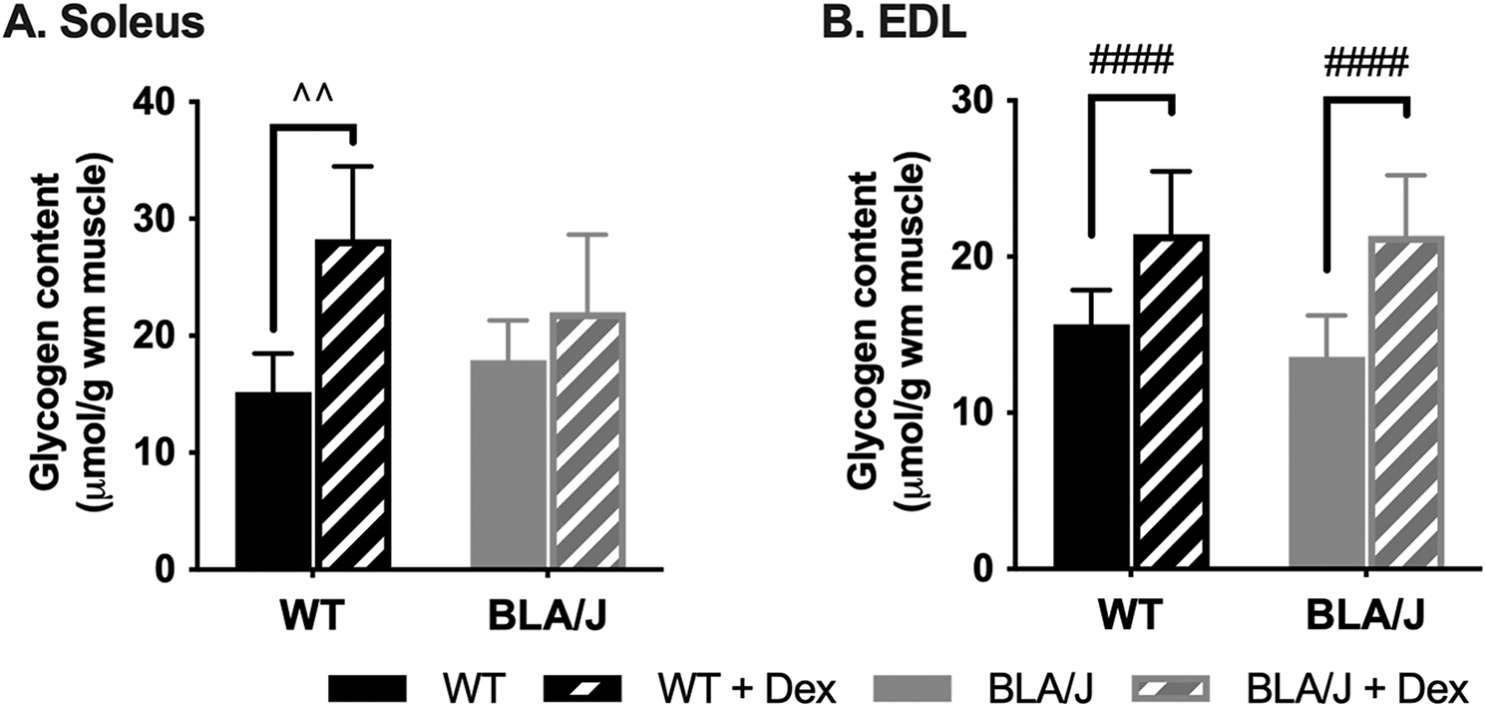
Glycogen content of (**A**) soleus and (**B**) EDL muscles of WT and dysferlin-deficient BLA/J mice aged 10 months, without and with dexamethasone (Dex) treatment. Whole muscle homogenates analysed for glycogen content. Data were analysed by two-way ANOVA: #### Dex-treated vs untreated (*p* < 0.0001, treatment effect). ^^ significant difference between groups (*p* < 0.01, strain/treatment interaction effect). Data are presented as mean ± SD (*n* = 6)

An initial quantification of glycogen levels for soleus of BLA/J mice (aged 10 months) showed that Dex treatment increased glycogen content by 4.32 µmol/g of muscle wet mass (WM) (BLA/J: 37.17 ± 1.01 µmol/gWM, BLA/J + Dex: 41.49 ± 1.40 µmol/gWM; Student’s t-test *p* < 0.05). Further to this initial glycogen result, prompted by the unexpected histological observation of high levels of glycogen in both untreated and Dex-treated BLA/J quadriceps and psoas muscles, additional quantification of glycogen was undertaken. The histological PAS staining does not use a calibrated system, so biochemical analyses where a standard curve enables quantification were then undertaken to determine glycogen levels in all the soleus and also EDL muscles (Fig. 8).

Dex increased glycogen content in WT soleus muscles (*p* < 0.01; Fig. 8A) and all EDL muscles (*p* < 0.0001; Fig. 8B). While Dex did not significantly increase glycogen in the BLA/J soleus, the difference between untreated and Dex-treated BLA/J soleus was an increase of 4.1 µmol/gWM, similar to that of the previous assay, although the variability in this second assay was greater. Thus, the relative increase in glycogen after Dex treatment for the BLA/J soleus is unclear.

## Discussion

Our studies focused on investigating the molecular basis for the adverse effects of GCs in dysferlinopathy patients by exposing BLA/J and WT male mice to short-term Dex treatment to identify early changes at two different ages. For 5- and 10-month-old mice, we assessed the impact of Dex treatment via measures of phenotype and expression of metabolism and immune-associated genes in the psoas muscle. For the older 10-month-old mice, we did gene analyses for quadriceps and psoas muscles and also examined the impact of Dex on the histopathology of quadriceps and psoas muscles. Furthermore, in the soleus and EDL muscles, we investigated the effects of Dex on contractile function, MyHC composition, levels of proteins related to Ca^2+^ handling and metabolism, and glycogen content.

### Overall impact of Dex administration on BLA/J mice

Overall, Dex had little impact on muscle function and many other parameters measured. However, Dex treatment had some interesting effects on dysferlin-deficient BLA/J mice. Dex resulted in some sustained activation of the immune gene response by Dex in BLA/J muscles, with altered expression of specific genes associated with complement and the inflammasome. An unexpected effect was that Dex appeared to increase glycogen content in 10-month-old muscles, indicated by PAS staining in BLA/J quads and psoas, plus quantification of glycogen in soleus and EDL muscles. These preliminary results point to promising new directions for future studies.

The parallel treatment of WT mice with GCs revealed that WT mice were generally more responsive to Dex than BLA/J mice with respect to muscle function and expression of some genes (that varied between mice aged 5 and 10 months). For other parameters, the response to Dex was similar for both strains: Dex did not cause conspicuous loss of body mass nor quadriceps mass in either strain at either age, yet at 10 months, Dex increased soleus mass in both strains, and there was a similar shift in MyHC isoforms for all soleus and EDL muscles. Implications of these observations are discussed below, but first, it is pertinent to compare Dex with different GCs and delivery regimes to provide essential context for the following discussion.

### Comparison of Dex with different GCs and delivery regimens

In humans, around 0.75-1 mg/kg/day GCs is widely used to treat muscular dystrophies; for example, for the clinical trial with dysferlinopathy subjects, 1 mg/kg deflazacort was administered daily for the first month followed by every second day from months two to six [34]. In dysferlin-deficient mice, one study used intraperitoneal injection (~ 7 am) of 1 mg/kg prednisone for 4 weeks, either daily or weekly [36]. Another study in dysferlin-deficient mice, used oral gavage of prednisolone at a high dose of 30 mg/kg for 5 days (acute) or 3 months (chronic) and compared this with the benefits of a new steroid called vamorolone (VBP15) [37]. A recent commentary provides further insight into clinical and pre-clinical trials using vamorolone in muscular dystrophies [39]. In contrast with these situations, Dex delivered in drinking water is available for many hours, especially during the nocturnal active phase for mice when water consumption and metabolism is highest (equivalent to daytime delivery in humans).

Since the amount of water, and hence Dex, consumption varied between groups in our study (although not measured for individual mice), here we compare the average estimated effective daily Dex dosage with widely used prednisone/prednisolone. The biological half-life of Dex in tissues is longer (36–72 h) compared to its half-life in plasma, which is similar to prednisone/prednisolone (~ 3 h) [46]. Thus, Dex may remain elevated for longer in tissues (compared with prednisone/prednisolone), with night-time consumption of Dex in drinking water adding to existing tissue levels. Moreover, Dex has a greater affinity for the GC receptor and is a much more potent activator (~ 8-fold) than prednisolone [46]. In Study 1 (with 0.5 µg/mL Dex), on average, WT mice weighed 34.5 g and drank 5.00 mL/day, whereas BLA/J weighed 32.5 g and drank ~ 3.26 mL/day; and in Study 2 (with 0.75 µg/mL Dex), body mass was not different between groups and averaged 32.78 g, and mice drank ~ 6.76 mL/day. Therefore, assuming Dex to be 8-fold more potent than prednisolone in this system, the average effective Dex dose was estimated to be functionally equivalent to studies with prednisolone doses of ~ 0.58 mg/kg/day and ~ 0.40 mg/kg/day for WT and BLA/J mice, respectively, in Study 1, and ~ 1.24 mg/kg/day for both strains in Study 2. Despite a possible lower dose of Dex in young BLA/J mice (Study 1), strong effects of Dex were still evident at this age (discussed later).

In addition, multiple isoforms of the GC receptor arise by alternate splicing and translation initiation mechanisms that can respond to specific GCs in subtly different ways [47]. Furthermore, different tissues (e.g., muscles and adipocytes) can exhibit differential responses to GCs (sometimes transactivation, other times transrepression) depending on what accessory proteins bind to the GC receptor and what co-factors interact with the activated receptor (reviewed in Ramamoorthy et al. [47] and Herbelet et al. [38]). The impact of dysferlin deficiency on GC receptor isoform expression and the associated availability of co-factors is not known, but it may differ between WT and BLA/J mice, as well as between skeletal muscles and adipocytes. Consequently, the differential responses to Dex observed between the tissues may reflect the range of glucocorticoid receptor isoforms resident within each particular muscle (and myofibre) type, and further analyses would be required to determine the glucocorticoid receptor isoforms present in each muscle type.

Our initial Study 1 (in 2015) was designed to investigate the mechanistic basis of the adverse effects of classic sustained GC treatment on dysferlin-deficient muscles. However, recent studies show that intermittent administration of prednisone once weekly, rather than daily, can produce many of the benefits but reduce the adverse effects of GCs in muscular dystrophies (reviewed by Quattrocelli et al. [33]). This is demonstrated for dysferlinopathy, where once weekly prednisone (intraperitoneal, 1 mg/kg for 4 weeks) did not induce atrophy nor adipogenesis and was associated with improved muscle function in mice aged 9 months [36]. This intermittent GC administration (or perhaps vamorolone) presents other strategies to consider for future steroid treatment of dysferlinopathy.

### Effects of Dex treatment on metabolism

#### Dex and metabolism-associated genes in BLA/J muscles

We previously reported strain-specific differences with the remodelling of lipid metabolism genes in the quadriceps muscle of 18-week-old BLA/J mice [26], where genes encoding regulatory proteins of sphingolipid metabolism were uniformly increased. Furthermore, some changes occurred in select genes that would predict increased triglyceride storage in lipid droplets contained within myofibres (i.e., *Pnpla2*, *Plin5*). In the present study, examining genes encoding proteins that regulate metabolism did not provide a clear picture of the effects of dysferlin deficiency nor Dex treatment. There were various effects of Dex on gene expression patterns in the psoas that were less pronounced in BLA/J mice (compared with WT), although patterns were not consistent between mice aged 5 and 10 months and these differed from the pattern of gene expression in quadriceps at 10 months.

In psoas muscles of young (5 months) untreated BLA/J mice (compared with WT), there was increased expression of lipogenic genes (i.e., *Lpl*, *Fasn*, *Acly*, and *Srebf1*) that contribute to increased lipid deposition in muscle. There were also increases in *Pparγ* and *Cebpδ* expression, both genes associated with adipogenesis and inflammation, which are typically lowly expressed in skeletal muscle [67]. This accords with high levels of *Cebpd* mRNA in dysferlin-deficient quadriceps muscle also reported for male BLA/J and female A/J mice aged 3 months (increased > 1.5-fold compared with WT), emphasising the early upregulation of this gene in dysferlin-deficient muscles [13]. GCs are known to upregulate *Cebp* isoforms in skeletal muscle, at least under septic conditions [68], consistent with the effect observed for *Cebpd* in this study. We surmise that these effects might reflect the previously observed increase in differentiation of muscle-resident fibroadipogenic progenitors (FAPs) that contribute to progressive adipogenic replacement and degeneration of dysferlin-deficient muscle, manifesting initially only in specific muscles such as the psoas and quadriceps [17]. Interestingly, these differences were not observed in the psoas nor quadriceps muscles of 10-month-old BLA/J compared with WT mice. While other studies show upregulated mRNA expression of enzymes involved in lipogenesis and triacylglycerol synthesis pathways in many muscles in very old (~ 2 years) BLA/J mice [19], the interpretation of these observations is confounded by significant adipocyte replacement of muscles at this age.

Dex increased *Pparγ* expression in quadriceps of both strains at 10 months, which accords with increased *Pparγ* expression in BLA/J quadriceps after daily prednisone treatment for 4 weeks [36]. Dex treatment also increased expression of *Acaca* (associated with lipogenesis) and decreased *Glut4* (involved in glucose uptake and glycogen deposition). Together, our data indicate that molecular remodelling of metabolic genes is most likely an early manifestation of dysferlinopathy in mice and is exacerbated by GC exposure. Larger effect sizes in response to Dex would be likely after extended exposure (> 4–5 weeks) or at higher doses, further exacerbating the adverse phenotype evident in BLA/J mice.

The reasons for these observations are unclear but may relate to the dosage and delivery regime for Dex and the different ages of mice in these studies. The mice were euthanised during the day (inactive phase) when Dex levels would be at their lowest because mice drink (and feed) mostly at night (active phase). Dex effects on glycogen and lipogenic gene expression are also heightened in fasted animals and blunted or absent in fed animals [69, 70]. Therefore, since most Dex exposure occurred in the dark phase, the fed state may have blunted effects on gene expression.

It is probable that Dex increased adipogenic conversion of muscle-resident dysferlin-deficient FAP cells, as indicated by others who assessed lipid droplet production in FAPs cultured from muscle [36]. However, subtle effects might not be detected using gene expression analysis in skeletal muscle because FAPs constitute only a small fraction of the cellular mass, with FAPs shown to increase with age in BLA/J muscles (compared with WT) up to only about 3% of muscle area by 18 months [17].

Notably, the fold changes in gene expression in the 5-month cohort are generally much larger than those in the 10-month cohort. For example, untreated 5-month-old BLA/J mice expressed between ~ 10 and 60-fold more of a given gene (Fig. 3), while the differences between 10-month-old WT and BLA/J are much smaller (~ 2-3-fold; Fig. 4). However, care needs to be taken with direct comparisons of fold changes since the studies were conducted in different cohorts and different facilities, and gene expression was analysed using different methodologies. Values in Study 1 should not be compared to values in Study 2; rather, the focus should be on patterns within rather than across studies, where there are substantial and consistent changes in the expression of given genes between groups in both 5 and 10-month-old cohorts.

#### Dex and adipogenesis in BLA/J mice

Of potential interest, a further observation in Study 1 was that Dex increased the epididymal fat pad mass about two-fold in BLA/J mice (compared with untreated), with no impact of Dex on WT (data not shown). However, this preliminary result requires future substantiation (since fat pads were not sampled in Study 2). In the untreated BLA/J mice at 5 months (Study 1), the epididymal fat pad mass was ~ 50% lower compared with WT mice (data not shown), consistent with our previous observations with MRI in mice aged 4 months, showing that epididymal and subcutaneous adipose weights were 71% and 61% lower in BLA/J mice, respectively [26]. The genotype-specific increase in adiposity after only 5 weeks of Dex treatment indicates a Dex-stimulated increase in dysferlin-deficient adipocyte number and size. This accords with observations that longer-term (3 months) treatment with daily prednisolone increased adipogenesis in dysferlin-deficient mouse muscle [37], whereas treatment with the steroid vamorolone reduced such adipocyte content. Clearly, it is of interest to comprehensively sample and analyse adipose tissue throughout the body in future GC studies.

This adipogenic effect of Dex and prednisolone might be accounted for by an increase in GC receptor [41] or enhanced post-receptor signalling in dysferlin-deficient adipocytes or FAPs [71]; FAPs are now considered to be similar or identical to fibroblasts since both can differentiate into either adipocytes or myofibroblasts. If this rapid GC effect on dysferlin-deficient adipocytes occurs in human muscles (as observed after 3 months in dysferlin-deficient mice [37]), then many months of clinical administration of GCs might exacerbate the replacement of dysferlin-deficient myofibres by dysferlin-deficient adipocytes, and contribute to the reported histopathology and loss of muscle function in humans with dysferlinopathy [33].

### Impact of Dex treatment on the immune system

#### Dex has varied effects on intrinsically high expression of complement-related genes in BLA/J muscles

In (untreated) BLA/J muscles (compared with WT), there was intrinsic high expression of genes associated with the complement cascade and membrane attack complex (MAC) formation, as well as genes related to the inflammasome (in both studies). Complement activation and the presence of MAC on many apparently ‘uninjured’ (non-necrotic) myofibres has been observed in dysferlinopathies, in contrast with the absence of these in many other myopathies [50, 72]. A clinical immunohistological study emphasised that MAC deposit was observed on the sarcolemma of non-necrotic myofibres only in dysferlinopathy [73]. Reduced decay-accelerating factor (DAF)/CD55 mRNA levels were also reported for human dysferlinopathic muscles, although this was also observed in other muscular dystrophies [74].

Perturbed regulation of the complement pathways was also reported in dysferlin-null mice [51], showing complement C5b-9 deposits on myofibres and, similar to our data, increased expression of complement-related genes in quadriceps muscles of BLA/J mice compared with WT mice. Furthermore, genetic ablation of C3 in dysferlin-deficient mice reduced disease severity, indicating that activation of C3 accelerates muscle damage in dysferlin-null mice and that complement-mediated injury is central to the pathogenesis of dysferlinopathy [51]. An earlier study identified increased susceptibility to complement attack due to down-regulation of DAF1/CD55 and DAF2/CD55b only in dysferlin-deficient muscles, demonstrated by mRNA and protein analyses in dysferlin-deficient mouse models and patients with dysferlinopathy [52]. Down-regulation of *Daf1* was also shown, via microarray, for 2 and 9-month-old dysferlin-deficient quadriceps [75]. In contrast, for the 5-month-old BLA/J psoas muscles, we did not observe decreased levels of *Daf1* (CD55) nor *Daf2* (CD55b), possibly explained by different muscles used for analyses or the targeted approach presented here.

While Dex treatment decreased the expression of many complement-related genes in WT psoas muscles at 5 months, consistent with its known potent anti-inflammatory properties, it had little impact on these genes in BLA/J psoas at this age. Precisely what attenuates this typical anti-inflammatory effect of Dex in BLA/J mice is unclear. At 10 months, there was no consistent pattern for quadriceps nor psoas muscles, although Dex increased *C4* in WT muscles; these different responses to Dex at the two ages are difficult to reconcile but might relate to the different duration and the effective dosage of Dex used (discussed above), which may effect the downstream protein measurements.

While GCs are typically considered to have anti-inflammatory effects (as evident for young WT psoas muscles in response to Dex treatment at 5 months), there is strong evidence that GCs can also stimulate the inflammatory response, as shown for normal human peripheral blood mononuclear cells (lymphocytes and monocytes) stimulated ex vivo with Dex. This treatment induces many inflammatory markers, including cytokines and complement components, but also represses the expression of adaptive immune-related genes, where the response can depend on whether the cell is resting or activated [76]. Since dysferlin-deficient muscles contain more macrophages than WT, and the dysferlin-deficient macrophages are disturbed [74], this might influence the overall response to Dex, especially since complement regulation at the mRNA level appears intrinsically perturbed by dysferlin deficiency. Our new gene expression data for BLA/J muscles aged 5 and 10 months and the published clinical and pre-clinical studies strongly support the involvement of the immune system, complement activation, and MAC in the subsequent death of dysferlin-deficient myofibres. Why dysferlin deficiency results in this early complement activation and MAC deposition on myofibres is currently unclear. However, it would be valuable to consider possible therapies that target the complement cascade: such targeted therapies are now of interest for many diseases with an increasing number of drugs available [77–79].

#### Impact on already high expression of inflammasome-related genes in BLA/J muscles

Two key genes related to the inflammasome, *Casp1* and *Nlrp3*, were highly expressed in BLA/J psoas muscle at 5 months (compared with WT), and both were also significantly increased by Dex in BLA/J but not WT muscle. Similarly, at 10 months, *Casp1* expression was elevated in BLA/J quadriceps and psoas muscles compared with WT, and *Nlrp3* was elevated in BLA/J psoas but not quadriceps. Dex treatment did not impact these genes in 10-month-old quadriceps and psoas muscles.

The NLRP3 inflammasome is activated by a range of factors, including sterile cell damage and complement [80]. Sublethal complement MAC formation (but not C3a and C5a) activated the NLRP3 inflammasome in vivo [81], which appears to involve C5aR2 and also C5a [82]. Activated NLRP3 drives chronic inflammation via caspase-1-mediated proteolytic cleavage and secretion of proinflammatory cytokines, interleukin-1β and interleukin-18 [83]. Our gene expression data and previous reports of high levels of MAC on dysferlin-deficient myofibres accord with observations that the inflammasome is strongly up-regulated in dysferlin-deficient mouse and human skeletal muscles and “may initiate, exacerbate, and possibly perpetuate the underlying myofibre-specific dystrophic process” [84].

Additionally, our data indicate that Dex further exacerbates this heightened inflammasome in the young BLA/J psoas muscles. In contrast with classic anti-inflammatory effects, Dex and other GCs can induce NLRP3 expression and hence the inflammasome, shown in mouse and human macrophages [85, 86]. In BLA/J muscles, there might also be some dysregulation of complement regulators, resulting in a limited response to Dex and consequent downstream activation of the inflammasome. A pro-inflammatory effect of Dex on the BLA/J inflammasome might have an additional impact on dysferlin-deficient macrophages since these are increased in dysferlin-deficient muscles and have enhanced phagocytic activity [74]. A better understanding of GC-mediated effects on dysferlin-deficient macrophages may help develop targeted therapies for different diseases [86] and be relevant to the complicating effects of dysferlin-deficient macrophages in skeletal muscles from dysferlin-deficient mice.

### Impact on histopathology and glycogen content of BLA/J quadriceps and psoas muscles

Dex-treated BLA/J quadriceps and psoas muscles had increased areas of adipocytes intruding into edges of some muscle sections, along with suggestion of additional isolated adipocytes between the myofibres. These observations accord with Dex stimulating adipogenesis but need to be substantiated in further studies, ideally using a longer duration of Dex treatment.

A more consistent result of Dex treatment was a range of ‘altered’ BLA/J myofibres, including many pale myofibres in H&E-stained sections, which were pronounced in the larger quadriceps muscles (but also evident in the psoas). The increased number of vacuoles in the pale sarcoplasm of many myofibres, especially for large pale myofibres, suggested swelling and possible myofibre death by oncosis, a term for cell death with swelling [87, 88]. Such swelling (with paler sarcoplasm) can suggest water uptake by individual myofibres. One reason for this is increased glycogen, since 1 g of glycogen binds to about 2.7 g of water and is known to increase the cross-sectional area of myofibres [65]. It is well documented that GC treatment can result in glycogen accumulation in skeletal muscles due to enhanced glucose uptake [69, 70, 89], and it is emphasised that age and health influence this response to GCs. One study in diabetic mice showed that the GC corticosterone (delivered in drinking water) doubled the amount of glycogen in the rectus muscle of male NOD mice after 6 weeks [90]. Moreover, muscle glycogen was increased in rats by 15 days of intraperitoneal Dex [70]. These combined data strongly support the possibility that Dex (for only 4 weeks) increased glycogen levels and myofibre swelling, resulting in oncosis in the severely affected myofibres in BLA/J muscles. In addition, since the sarcolemma of dysferlin-deficient myofibres is shown to be weakened by hypo-osmotic shock injury in tissue culture (due to abnormal RyR1 Ca^2+^ leaks) [91, 92], this vulnerable dysferlin-deficient sarcolemma may exacerbate the pronounced swelling of BLA/J myofibres and further contribute to oncosis in vivo. This novel possibility for death of dysferlin-deficient myofibres, warrants further studies for dysferlin-deficient human and mouse muscles.

The mechanistic metabolic basis for the high glycogen evident in many myofibres in the BLA/J limb-girdle muscles with marked histopathology (but not conspicuous in muscles with minimal histopathology, e.g., soleus and EDL) requires dedicated experimental investigation. Of particular interest is the extent to which this high glycogen is a cause, or a consequence, of myofibre damage and dystropathology. Metabolic changes reported in dysferlinopathy have largely focused on lipids [26].

### Dex has limited effect on BLA/J soleus and EDL muscles

Generally, for the 10-month-old mice, the BLA/J soleus showed more changes than EDL, and Dex had minimal effects, but these were more pronounced for the soleus. One feature of interest is that BLA/J soleus muscles were heavier than WT muscles at 10 months, possibly due to increased oedema (discussed in Lloyd et al. [55]). Whether oedema, perhaps linked with increased glycogen content [93], may also be implicated in the specific response to Dex of soleus muscles (i.e., increased mass for both strains) is unclear and remains to be investigated.

#### Muscle function

For untreated 10-month-old male mice, our ex vivo studies show subtle, muscle-specific alterations in the contractile function of BLA/J muscles (compared with WT), with the soleus muscle showing more changes than EDL; these results have been discussed in detail in Lloyd et al. [55]. Dex had some effects on muscle function, mainly in WT mice, with increased grip strength, ex vivo soleus tetanic force, faster twitch contraction times, and slower post-fatigue recovery. In WT EDL, there was slower twitch relaxation time, reduced fatigue, and improved post-fatigue recovery, as reported previously [94, 95]. In contrast, there were no BLA/J-specific effects of Dex treatment on muscle performance: BLA/J soleus and EDL muscles appeared largely refractory to Dex and were only impacted by overall effects (i.e., regardless of strain), with a faster rate of force production for Dex-treated soleus and slower time to peak twitch in Dex-treated EDL. Since these muscles have relatively little dystropathology, it is relevant for future studies to instead utilise functional measurements that more directly assess the problems with severely affected BLA/J limb-girdle muscles, using approaches such as analyses of gait and walking along a balance beam [15].

A study in dysferlin-deficient mice (aged 9 months) using intraperitoneal 1 mg/kg prednisone for 4 weeks showed reduced body and muscle mass and loss of in vivo muscle strength from daily GC administration [36]. This was in contrast to the benefits of once-weekly dosing, which included increased myofibre cross-sectional area, grip strength, and maximum force production [36]. Presumably, such differences between studies reflect the GC regimes used and perhaps the background strains for the dysferlin-deficient mice.

#### MyHC composition and abundance of proteins

Myofibre-type composition of soleus and EDL muscles for untreated WT and BLA/J mice have already been described [55]. Dex had similar effects on myofibre-type proportions in both strains, with an increased abundance of MyHC 2X protein in soleus and EDL muscles and a concomitant decrease in MyHC 2B in EDL. The functional effects of this may be more evident in the WT soleus with a lower proportion of type 2X myofibres initially and may explain the quicker contraction and relaxation in the Dex-treated WT soleus compared with untreated. This shift in MyHC composition in the Dex-treated soleus, away from slow-twitch fatigue-resistant type 1 myofibres, is consistent with reports of faster contraction times and increased fatigability in soleus muscles of GC-treated rats [95]. In contrast, Dex-treatment of EDL muscles decreased the abundance of fast type 2B myofibres and increased the abundance of intermediate type 2X myofibres. This likely explains the slower contraction (for WT and BLA/J) and relaxation (WT only) of the Dex-treated EDL, consistent with functional studies in rabbit EDL muscles [94]. As mentioned, since Dex had no impact on the levels of proteins related to Ca^2+^ handling and metabolism, this aspect is not discussed further here.

#### Glycogen content

We previously showed that dysferlin deficiency did not impact glycogen content of whole 10-month-old soleus and EDL muscles, although BLA/J soleus muscles had more glycogen than BLA/J EDL muscles [61]. Our current studies show that Dex significantly increased the glycogen content of WT soleus and both WT and BLA/J EDL muscles; however, the extent to which Dex increased glycogen in BLA/J soleus was unclear and requires further clarification.

These combined studies emphasise the value of comparing predominantly slow (soleus) and fast (EDL) muscles to critically ascertain different consequences of dysferlin deficiency, especially across species where the myofibre composition of different muscles can vary [57]. Such myofibre-type influence needs to be considered when extrapolating data for observations derived from muscles with different myofibre-type composition; for example, the widely used mouse quadriceps (~ 59% fast-twitch glycolytic type 2B) compared with human biopsies, often from vastus lateralis (~ 3–16% fast-twitch type 2X: the most glycolytic myofibre in human muscles) [57], where the overall myofibre composition may obscure the differential impact of dysferlin deficiency.

### Conclusions and future studies

Our in vivo studies showed remarkably little impact of short-term administration of the GC Dex on dysferlin-deficient BLA/J (compared with WT) mice at two ages, except for three potential aspects: (i) stimulation of adipogenesis, (ii) increased inflammasome that will further damage dysferlin-deficient muscles, and (iii) increased glycogen content with possible myofibre death by oncosis. We recognise that these are preliminary observations but they highlight new areas for study to (i) identify the mechanistic basis of dystropathology in dysferlin-deficient muscles and (ii) gain further insight into the unexpected adverse effects of GCs in dysferlinopathy. These observations also emphasise the need to investigate levels of GC receptors and associated signalling molecules in different dysferlin-deficient muscles and adipocytes, ideally also using RNA Seq and other broad analytical tools.

Future studies investigating GC treatment must carefully consider the type of GC and regime used for administration because the use of different regimes (e.g., time of day or night, frequency) and modes of delivery (e.g., in food/water, by gavage or injection) in animal models, complicates the widely reported benefits and adverse effects of GCs on muscular dystrophies. Thus, it is prudent to evaluate the implications of these variables in future experimental and clinical designs to investigate the long-term use of GCs in dysferlinopathy.

### Electronic supplementary material

Below is the link to the electronic supplementary material.

Supplementary Material 1

## Data Availability

Data are available from the corresponding author upon reasonable request.
