# Supplementary material for: Pilot investigations into the mechanistic basis for adverse effects of glucocorticoids in dysferlinopathy
Source: Skelet Muscle. 2024 Aug 9;14:19. doi: 10.1186/s13395-024-00350-6 (PMC11312411; doi:10.1186/s13395-024-00350-6)
Supplement: Supplementary file 1 — Supplementary Material 1 [file 13395_2024_350_MOESM1_ESM.doc]

**Supplementary Material**

**Supplementary Table 1: List of genes quantified by qPCR.**

| **Gene** | **Encoding protein** | **Primer sequence or Qiagen catalogue number** | |
| --- | --- | --- | --- |
|  |  | **Study 1 (young)** | **Study 2 (old)** |
| ***Metabolic and lipogenic/adipogenic genes*** | | | |
| *Acaca* | Acetyl-CoA carboxylase 1 | F5’ CTGTATGAGAAAGGCTATGTG 3’  R5’ AACCTGTCTGAAGAGGTTAG 3’ | QT00258419 |
| *Acly* | ATP citrate lyase | F5’ ATATTCATCAGCTTCCTCCC 3’  R5’ TCCAAGAAGCCAAATCTTATCCTG 3’ | QT00163030 |
| *Cebpa* | CCAAT enhancer binding protein alpha | F5’ AAGGGTGTATGTAGTAGTGG 3’  R5’ AAAAAGAAGAGAAGGAAGCG 3’ | QT00311731 |
| *Cebpδ* | CCAAT/enhancer-binding protein delta | F5’ ACAAAGTGTTTAGGTTGGAC 3’  R5’ GTAAAGCTTCAGCCAGTATC 3’ | QT00295883 |
| *Chrebp* | Carbohydrate-responsive element-binding protein | - | QT00125335 |
| *Fasn* | Fatty acid synthase | F5’ GATTCAGGGAGTGGATATTG 3’  R5’ CATTCAGAATTCGTGGCATAG 3’ | QT00149240 |
| *Glut4 (Slc2a4)* | Glucose transporter type 4 | F5’ CAATGGTTGGGAAGGAAAG 3’  R5’AATGAGTATCTCATAGGAGGC 3’ | QT01044946 |
| *Lpl* | Lipoprotein lipase | F5’ GAGACTCAGAAAAAGGTCATC 3’  R5’ GTCTTCAAAGAACTCAGATGC 3’ | QT01046017 |
| *Pparγ* | Peroxisome proliferator activated receptor gamma | F5’ AAAGACAACGGACAAATCAC 3’  R5’ GGGATATTTTTGGCATACTCTG 3’ | QT00100296 |
| *Srebf1* | Sterol regulatory element-binding transcription factor 1 | F5’ AATAAATCTGCTGTCTTGCG 3’  R5’ CCTTCAGTGATTTGCTTTTG 3’ | QT00167055 |
| ***Immune and complement genes*** | | | |
| *C1qb* | Complement C1q B chain | QT00495299 | QT00495299 |
| *C3* | Complement component 3 | QT00109270 | QT00109270 |
| *C3ar1* | complement 3a receptor 1 | QT00251216 | - |
| *C4* | Complement component 4 | QT01074948 | QT01074948 |
| *C5* | Complement component 5 | QT00102032 | - |
| *C5ar1* | Complement 5a receptor 1 | - | QT00288232 |
| *C5ar2* | Complement 5a receptor 2 | QT00287553 | - |
| *Casp1* | Caspase 1 | QT00199458 | QT00199458 |
| *Daf1* | Decay-accelerating factor 1 for complement (CD55) | QT00133994 | - |
| *Daf2* | Decay-accelerating factor 2 for complement B (CD55b) | QT00172984 | - |
| *Nlrp3* | NLR family pyrin domain containing 3 | QT00122458 | QT00122458 |
| *Tnf* | Tumour necrosis factor | - | QT00104006 |
| ***Reference genes*** | | | |
| *Acta1* | Actin, alpha 1, skeletal actin | F5’ATTCCTTCGTGACCACAGCTGAACGT 3’ | - |
| *Hprt* | Hypoxanthine guanine phosphoribosyl transferase | R5’ CGCGAACGCAGACGCGAGTGCGC 3’ | - |
| *Ppia* | Peptidylprolyl isomerase A | F5’ AGGGATTTGAATCACGTTTG 3’ | F5’ AGCATACAGGTCCTGGCATC 3’ |
| *Sdha* | Succinate dehydrogenase complex flavoprotein subunit A | R5’ TTTACTGGCAACATCAACAG 3’ | R5’ TTCACCTTCCCAAAGACCAC 3’ |
| *Tbp* | TATA-box binding protein | - | F5’ TGGGGCGACTCGTGGCTTTC 3’ |
| Note: Some genes were only analysed in one study: - indicates not measured. | | | |

***Food and water consumption of young adult mice (4–5 months; Study 1)***

**
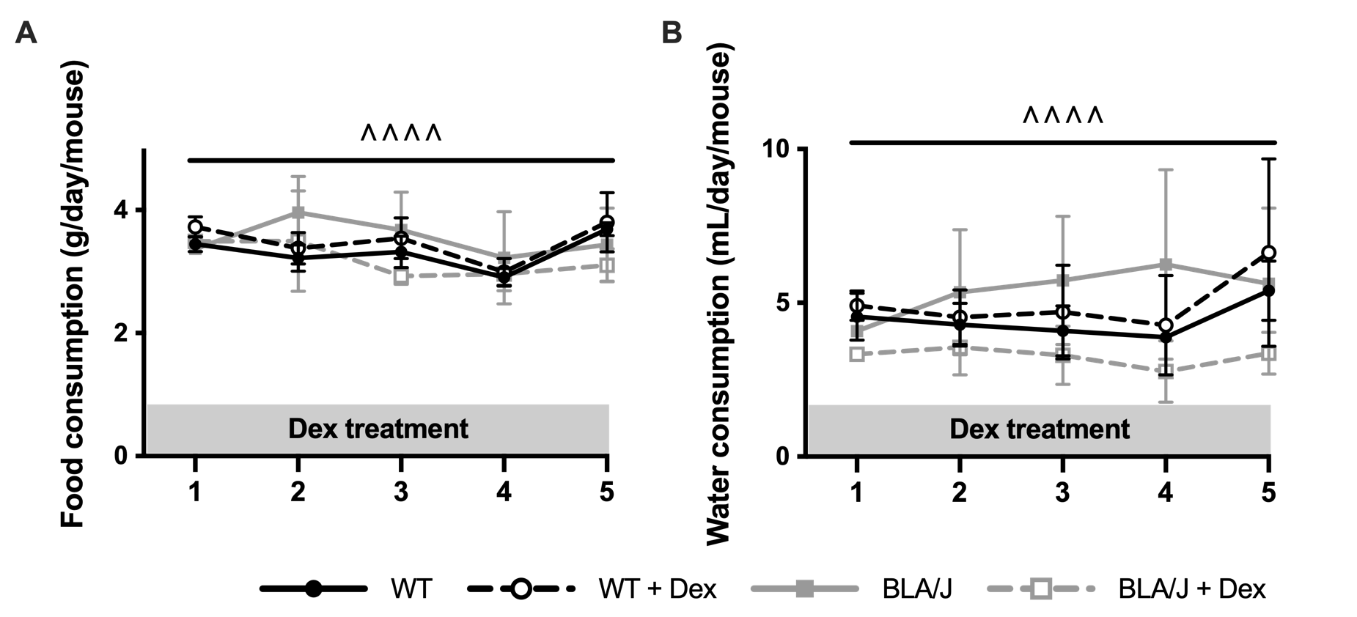
**

**Supplementary Fig. 1: Food and water consumption in groups of WT and dysferlin-deficient BLA/J mice aged 4–5 months, without and with dexamethasone (Dex) treatment.** (A) Food (g/day/mouse) and (B) water consumption (mL/day/mouse) over the 5-week treatment period. Data were analysed by 3-way ANOVA: ^^^^ BLA/J + Dex vs BLA/J untreated (*p* < 0.0001, strain/treatment interaction effect). Data are presented as mean ± SD (*n* = 8–9).

***Liver gene expression (5 and 10 months of age; Studies 1 and 2)***


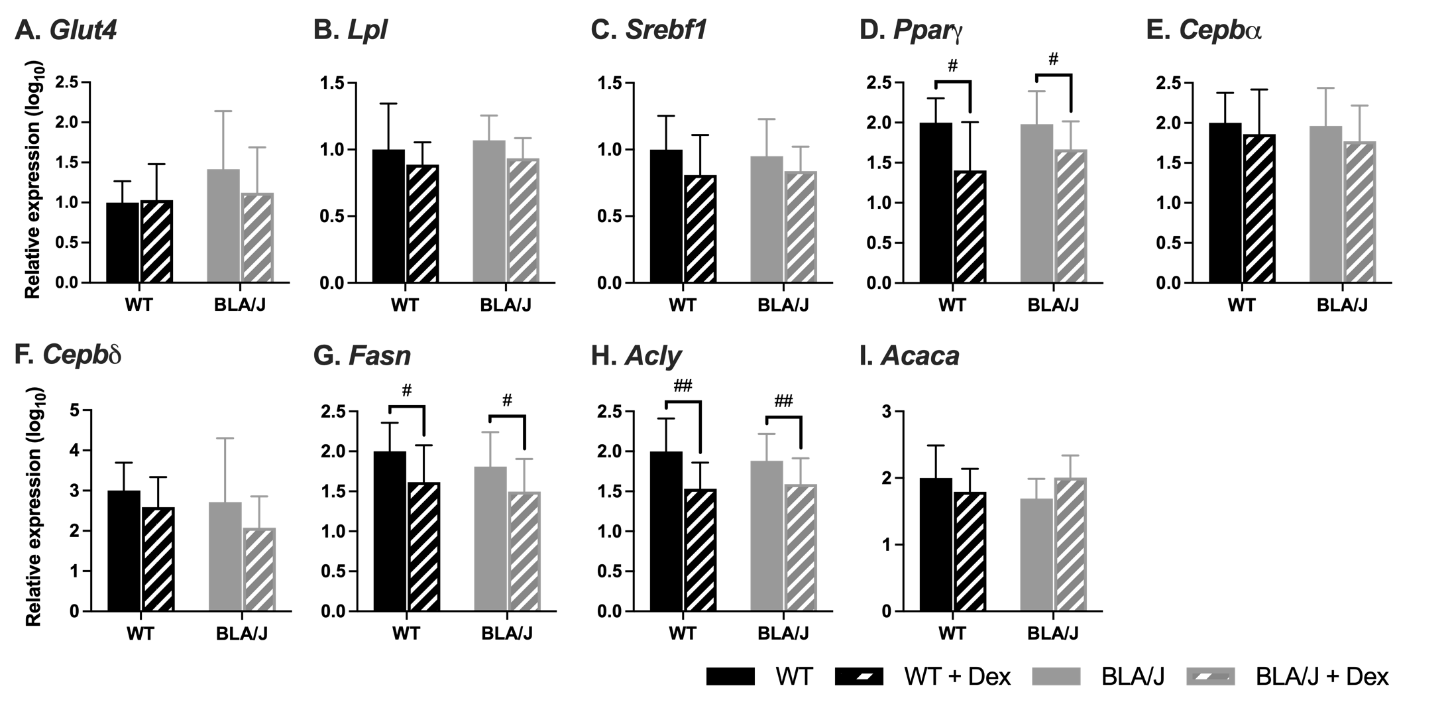


**Supplementary Fig. 2: Relative metabolism-associated gene expression in the liver of wild-type (WT) C57Bl/6J and dysferlin-deficient BLA/J mice aged 5 months, without and with dexamethasone (Dex) treatment.** (A) Glucose transporter type 4 (*Glut4*); (B) Lipoprotein lipase (*Lpl*); (C) Sterol regulatory element-binding transcription factor 1 (*Srebf1*); (D) Peroxisome proliferator-activated receptor gamma (*Pparγ*); (E) CCAAT enhancer binding protein alpha (*Cebpα*); (F) CCAAT/enhancer-binding protein delta (*Cepbδ*); (G) Fatty Acid Synthase (*Fasn*); (H) ATP citrate lyase (*Acly*); (I) Acetyl-CoA carboxylase 1 (*Acaca*). Hypoxanthine-guanine phosphoribosyltransferase (*Hprt*) was used as a reference gene to normalise gene expression values in the liver. Relative gene expression was calculated using the 2^−ΔΔCT^ Method, and values were normalised to the untreated WT for each gene. Data were analysed by two-way ANOVA: #, ## Dex-treated vs untreated *(p* < 0.05, 0.01, respectively, treatment effect). Data are log_10_ transformed and presented on a log_10_ *y*-axis scale as mean ± SD (*n* = 5–8).


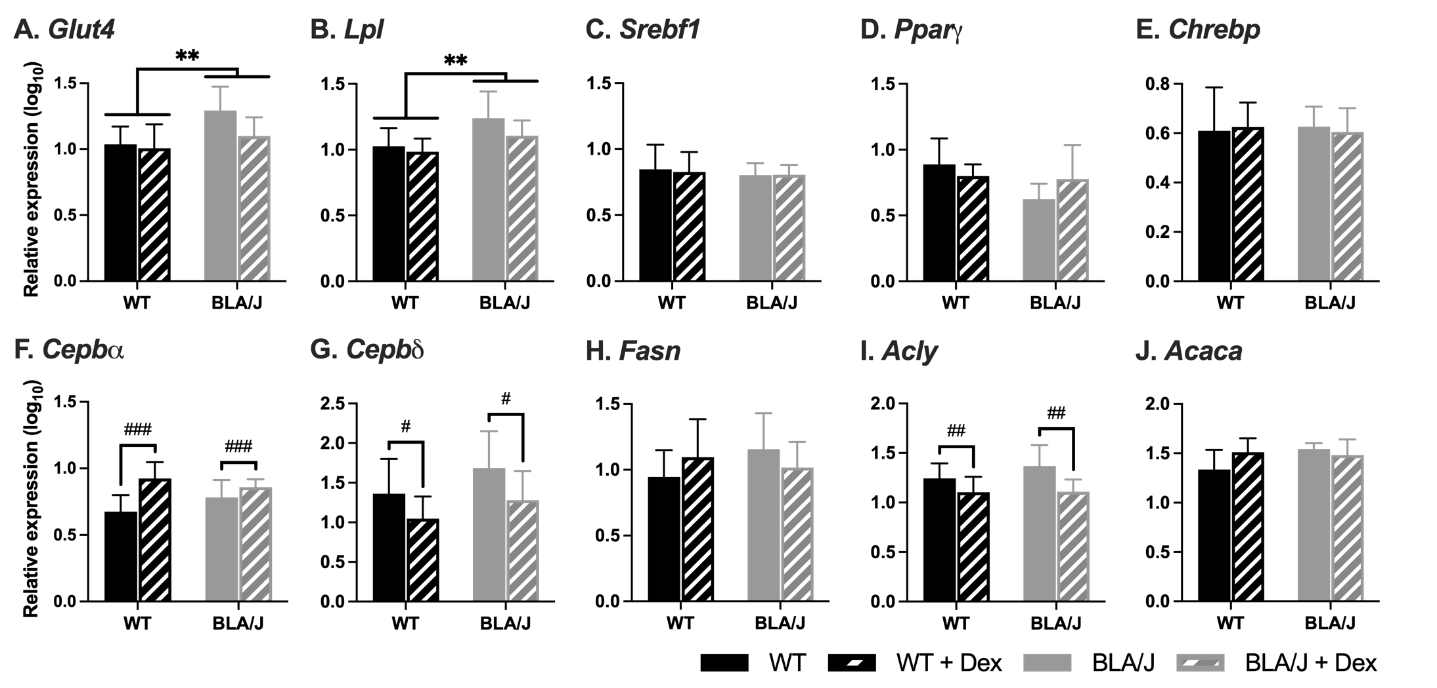


**Supplementary Fig. 3: Relative metabolism-associated gene expression in the liver of wild-type (WT) C57Bl/6J and dysferlin-deficient BLA/J mice aged 10 months, without and with dexamethasone (Dex) treatment.** (A) Glucose transporter type 4 (*Glut4*); (B) Lipoprotein lipase (*Lpl*); (C) Sterol regulatory element-binding transcription factor 1 (*Srebf1*); (D) Peroxisome proliferator-activated receptor gamma (*Pparγ*); (E) Carbohydrate-responsive element-binding protein (*Chrebp*); (F) CCAAT enhancer binding protein alpha (*Cebpα*); (G) CCAAT/enhancer-binding protein delta (*Cepbδ*); (H) Fatty Acid Synthase (*Fasn*); (I) ATP citrate lyase (*Acly*); (J) Acetyl-CoA carboxylase 1 (*Acaca*). All mRNA expression values were standardised against the reference genes peptidylprolyl isomerase A (*Ppia*) and TATA-box binding protein (*Tbp1*) using the GeNorm algorithm (64). Data were analysed by two-way ANOVA: ** BLA/J (± Dex) vs WT (± Dex) (*p* < 0.01, strain effect); #, ##, ### Dex-treated vs untreated (*p* < 0.05, 0.01, 0.001, respectively, treatment effect). Data are log_10_ transformed and presented on a log_10_ *y*-axis scale as mean ± SD (*n* = 7).


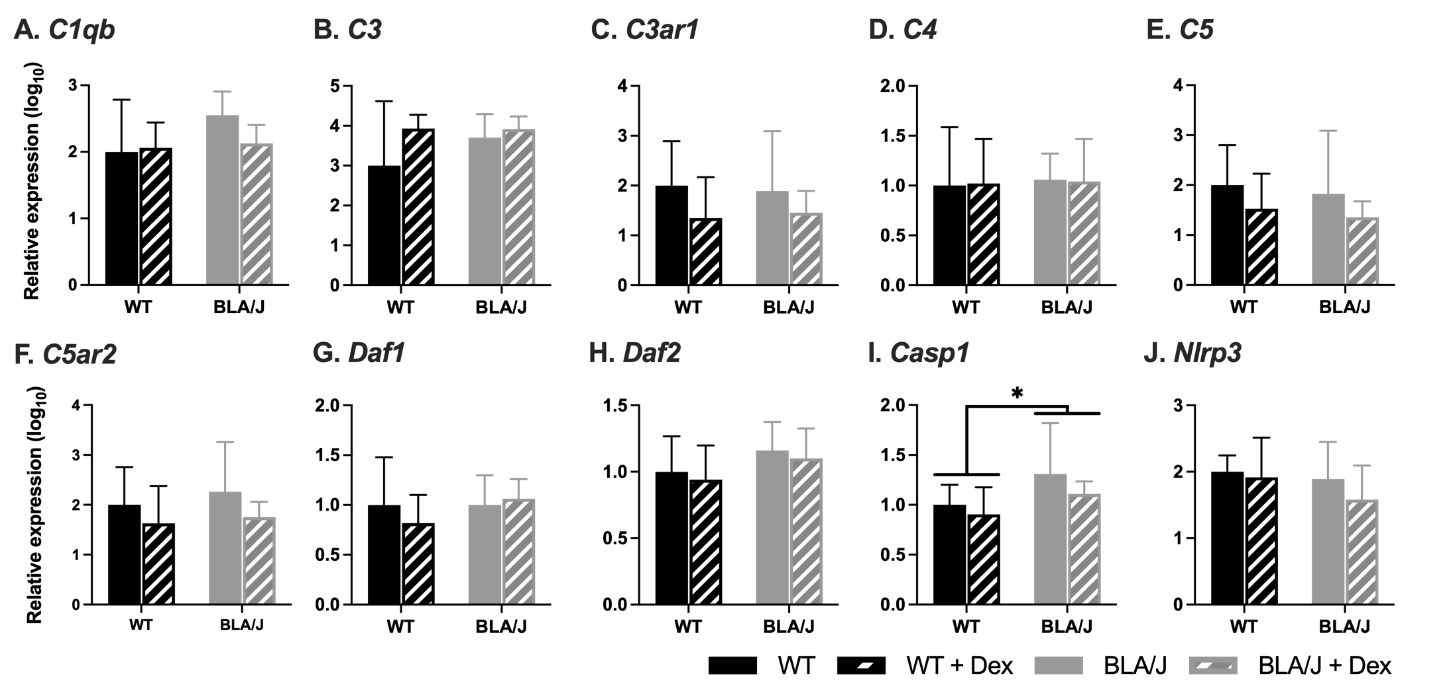


**Supplementary Fig. 4: Relative immune-associated gene expression in the liver of wild-type (WT) C57Bl/6J and dysferlin-deficient BLA/J mice aged 5 months, without and with dexamethasone (Dex) treatment.** (A) Complement C1q B Chain (*C1qb*); (B) Complement component 3 (*C3*); (C) Complement C3a Receptor 1 (*C3ar1*); (D) Complement component 4 (*C4*); (E) Complement component 5 (C5); (F) Complement C5a Receptor 2 (*C5ar2*); (G) Decay-accelerating factor 1 for complement (*Daf1* or CD55); (H) Decay-accelerating factor 2 for complement B (*Daf2* or CD55b); (I) Caspase 1 (*Casp1*); (J) NLR family pyrin domain containing 3 (*Nlrp3*). Hypoxanthine-guanine phosphoribosyltransferase (*Hprt*) was used as a reference gene to normalise gene expression values in the liver. Relative gene expression was calculated using the 2^−ΔΔCT^ Method, and values were normalised to the untreated WT for each gene. Data were analysed by two-way ANOVA: * BLA/J (± Dex) vs WT (± Dex) (*p* < 0.05, strain effect). Data are log_10_ transformed and presented on a log_10_ *y*-axis scale as mean ± SD (*n* = 5–8).


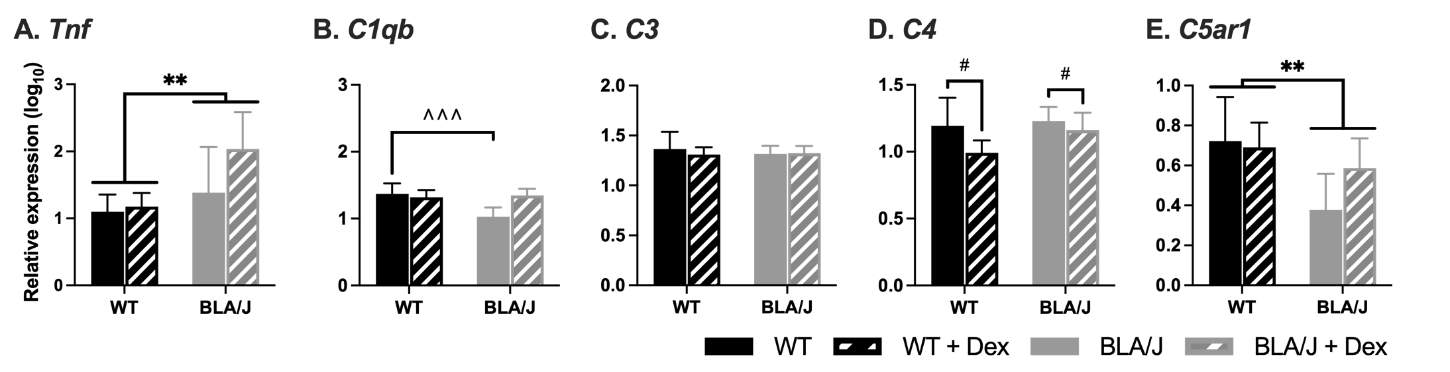


**Supplementary Fig. 5: Relative immune-related gene expression in the liver of wild-type (WT) C57Bl/6J and dysferlin-deficient BLA/J mice aged 10 months, without and with dexamethasone (Dex) treatment.** (A) Tumour necrosis factor (*Tnf*); (B) Complement C1q B Chain (*C1qb*); (C) Complement component 3 (*C3*); (D) Complement component 4 (*C4*); (E) Complement C5a Receptor 1 (*C5ar1*). All mRNA expression values were standardised against the reference genes peptidylprolyl isomerase A (*Ppia*) and TATA-box binding protein (*Tbp1*) using the GeNorm algorithm (64). Data were analysed by two-way ANOVA: ** BLA/J (± Dex) vs WT (± Dex) (*p* < 0.01, strain effect); # Dex-treated vs untreated (*p* < 0.05, treatment effect).; ^^^ significant difference between groups (*p* < 0.001, strain/treatment interaction effect). Data are log_10_ transformed and presented on a log_10_ *y*-axis scale as mean ± SD (*n* = 4–10).

***Other impacts of Dex treatment in older adult WT and BLA/J mice (10 months; Study 2)***

Muscle histopathology: quadriceps and psoas


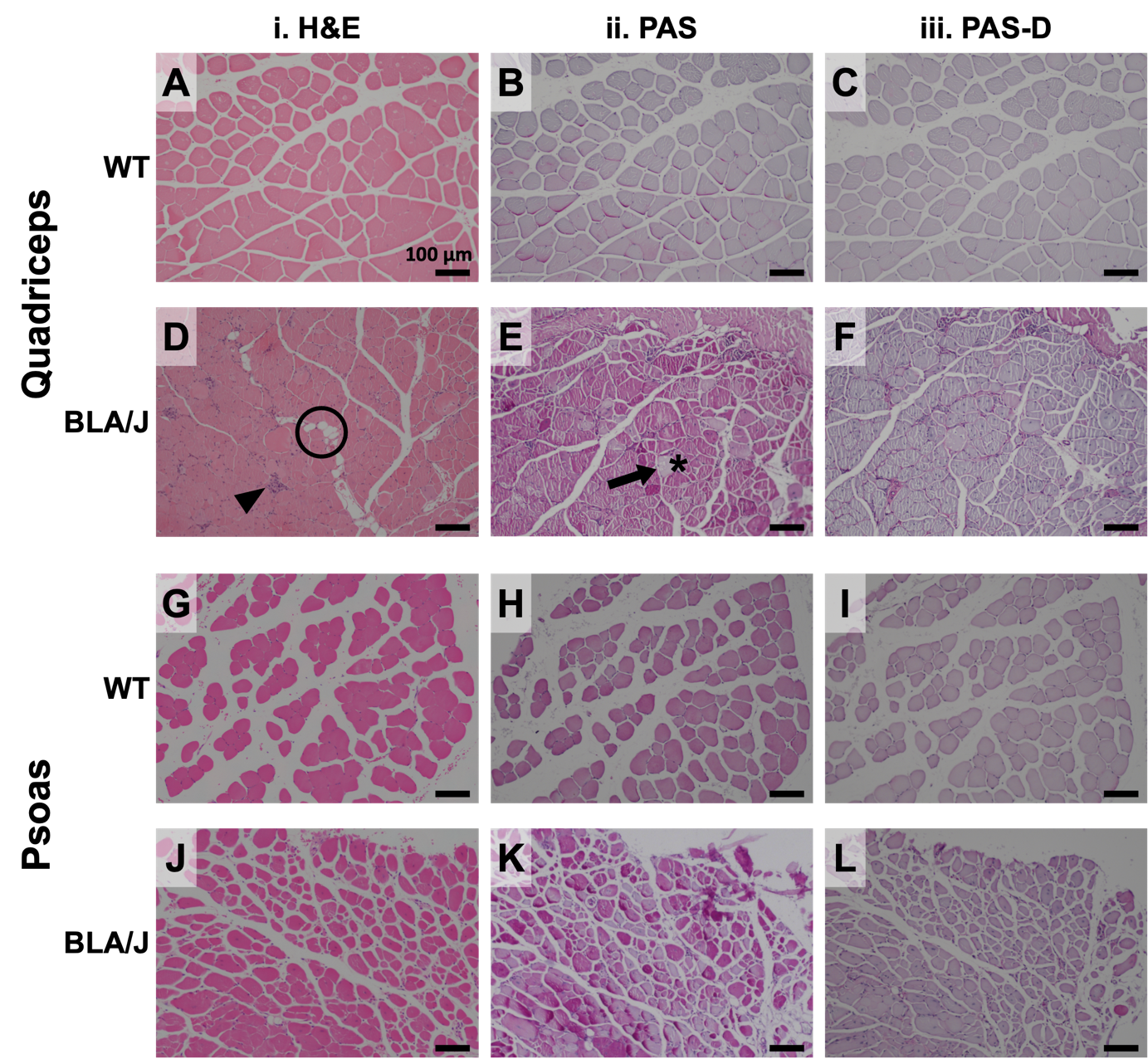


**Supplementary Fig. 6: Comparison of histopathology and glycogen staining for untreated WT and BLA/J quadriceps and psoas muscles (aged 10 months).** Representative images of paraffin sections showing (i) histopathology by haematoxylin and eosin (H&E), (ii) presence of glycogen by periodic acid-Schiff (PAS), and (iii) absence of glycogen following diastase-induced breakdown (as a control to verify glycogen PAS staining; PAS-D). Histopathological features include variable myofibre sizes, some adipocytes (circled), foci of inflammatory cells (arrowhead), and large pale myofibres (asterisks, *) with conspicuous vacuoles/fragmentation (arrow). Shown for 10-month-old male mice: WT (ID 16/39) and BLA/J (ID 16/25) quadriceps and WT (ID 16/38) and BLA/J (16/21) psoas. Scale bar = 100 μm.


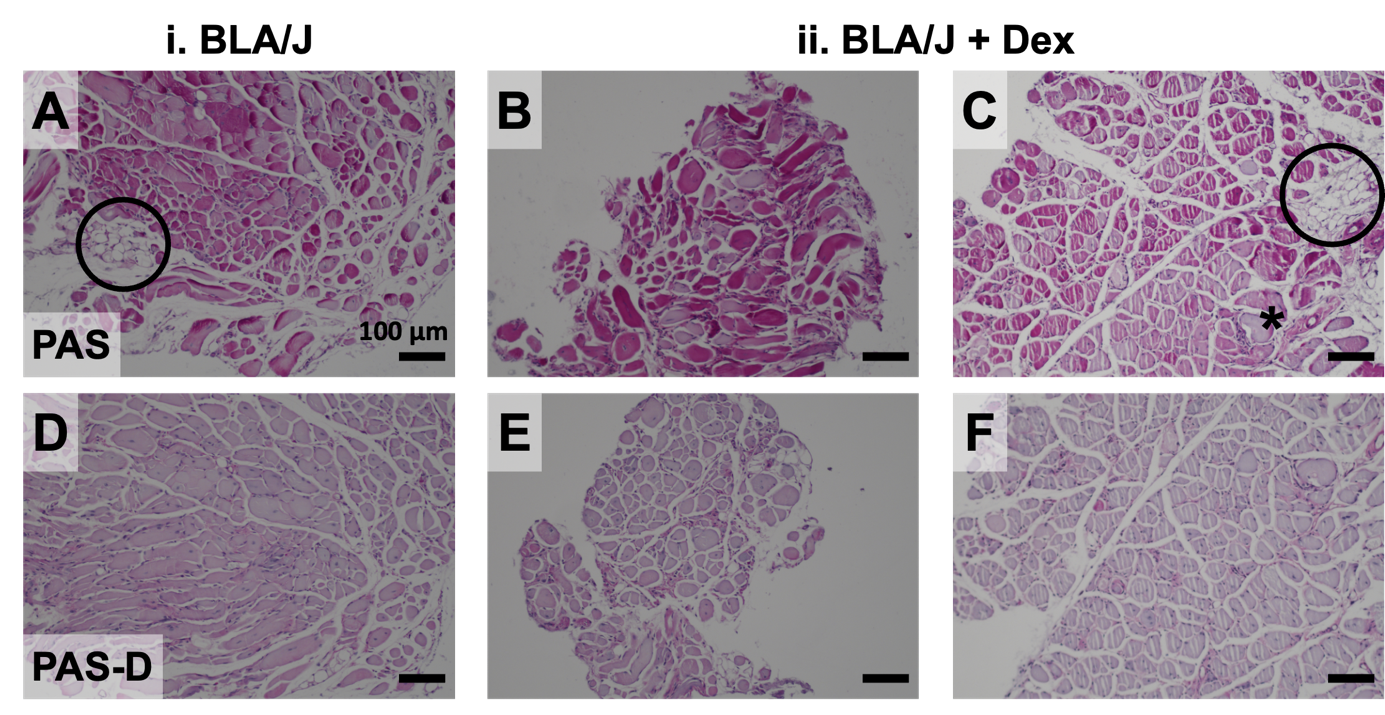


**Supplementary Fig. 7: Effect of dexamethasone (Dex) treatment on histopathology of psoas muscles of BLA/J mice (aged 10 months).** Representative images of paraffin sections showing highly variable histopathology of (i) BLA/J and (ii) BLA/J + Dex psoas by the (A-C) presence of glycogen by periodic acid-Schiff (PAS), and (D-E) absence of glycogen following diastase-induced breakdown (as a control to verify glycogen PAS staining; PAS-D). Histopathological features variable myofibre sizes, and some adipocytes (circled) and large pale myofibres (asterisks, *). Shown for three 10-month-old male mice: (i) BLA/J (ID for panels A, D. 16/21) and (ii) BLA/J + Dex (ID for panels B, E. 16/28; C, F. 16.33). Scale bar = 100 μm.


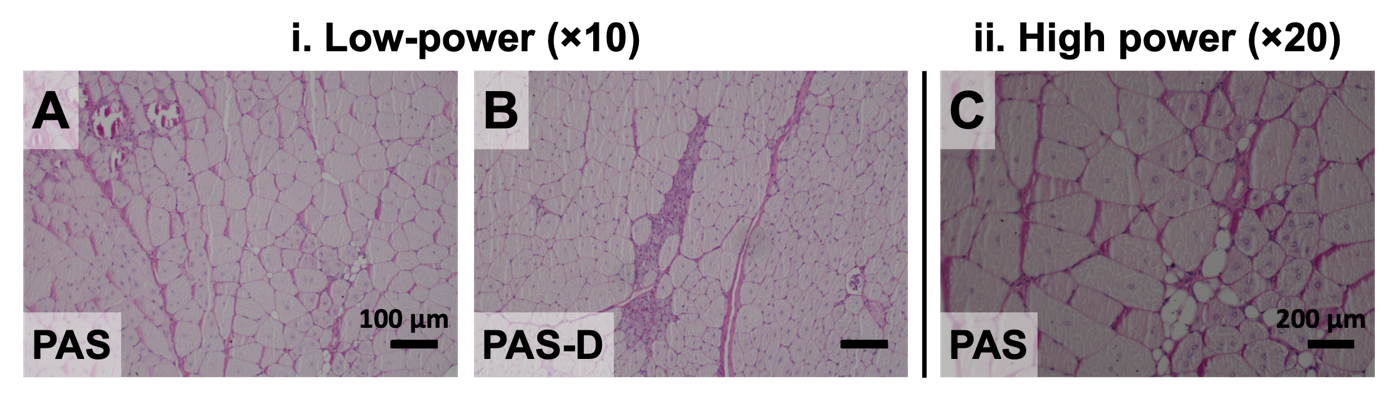


**Supplementary Fig. 8: Glycogen staining in dystrophic (untreated) *mdx* quadriceps muscles (aged 2 months) at (i) low power (×10) and (ii) high-power (×20).** Representative images of paraffin sections showing (A, C) glycogen staining by periodic acid-Schiff (PAS), and (B) absence of glycogen following diastase-induced breakdown (as a control to verify glycogen PAS staining; PAS-D). All images show many myofibres with central myonuclei, indicating regenerated myofibres that result from ongoing muscle necrosis in *mdx* mice. The low-power views include (A) an area of fragmented myofibres reflecting likely transient calcification and (B) a large region of recent muscle necrosis with many small new regenerating basophilic myofibres and small myofibres. Despite this pronounced pathology in *mdx* muscles, there is essentially no staining for glycogen. Scale bars = (i) 100 μm, (ii) 200 μm.

Muscle function: *in vivo* grip strength, *ex vivo* soleus and EDL contractile function and MyHC composition

**
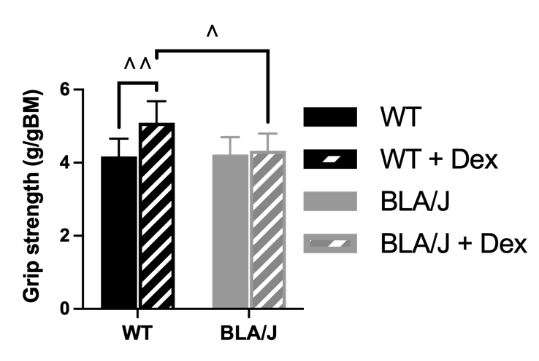
**

**Supplementary Fig. 9: Grip strength of WT and dysferlin-deficient BLA/J mice aged 10 months, without and with dexamethasone (Dex) treatment.** Grip strength was recorded on the day of sampling and normalised to body mass. Data were analysed by two-way ANOVA: ^, ^^ significant difference between groups (*p* < 0.05, 0.01, respectively, strain/treatment interaction effect). Data are presented as mean ± SD (*n* = 8–9). *Note: the data for untreated WT and BLA/J muscles has been published previously (55).*

**
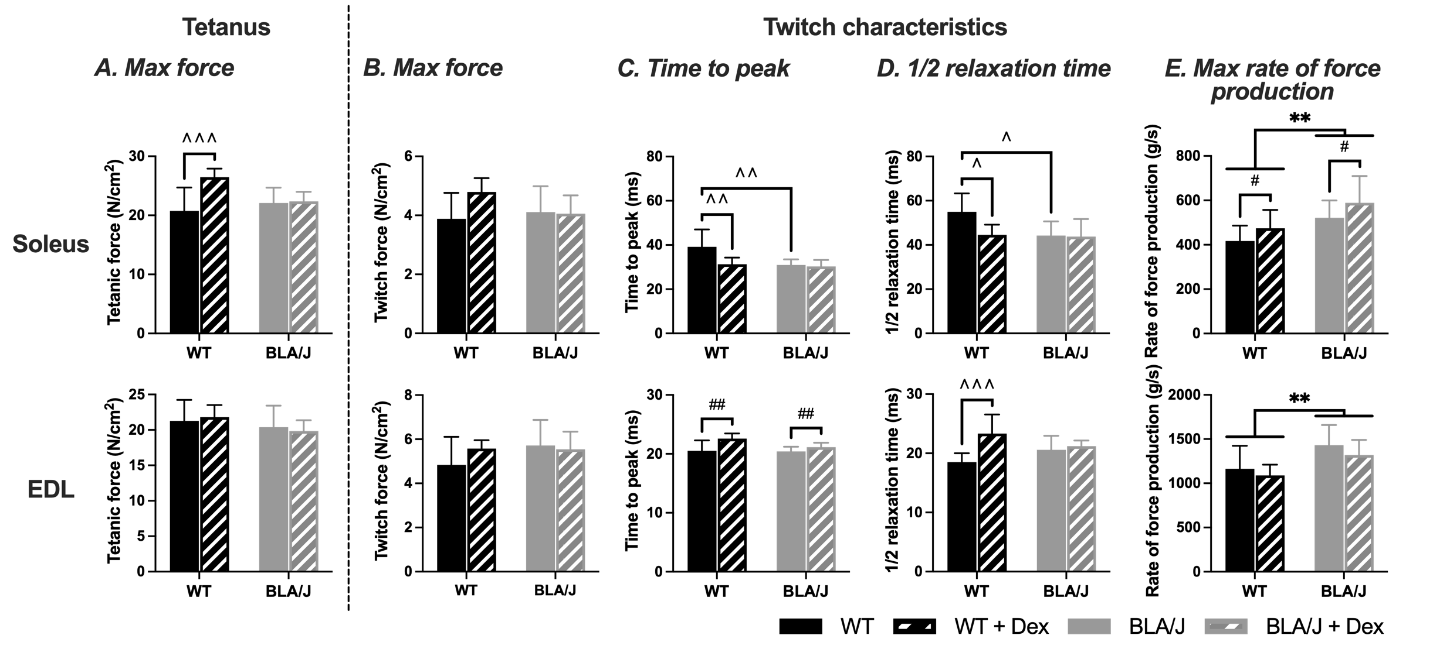
**

**Supplementary Fig. 10: *Ex vivo* measurements of contractile function for soleus and EDL muscles of WT and dysferlin-deficient BLA/J mice aged 10 months, without and with dexamethasone (Dex) treatment.** (A) Maximum tetanic force, (B) maximum twitch force, (C) time to peak twitch force, (D) ½ relaxation time, and (E) maximum rate of force production. Data were analysed by two-way ANOVA: ** BLA/J (± Dex) vs WT (± Dex) (*p* < 0.01, strain effect); #, ## Dex-treated vs untreated (*p* < 0.05, 0.01, treatment effect); ^, ^^, ^^^ significant difference between groups (*p* < 0.05, 0.01, 0.001, respectively, strain/treatment interaction effect). Data are presented as mean ± SD (*n* = 8–9). *Note: The y-axis scale differs between the comparison of soleus and EDL due to their different functional properties. Note: the data for untreated WT and BLA/J muscles has been published previously (55).*


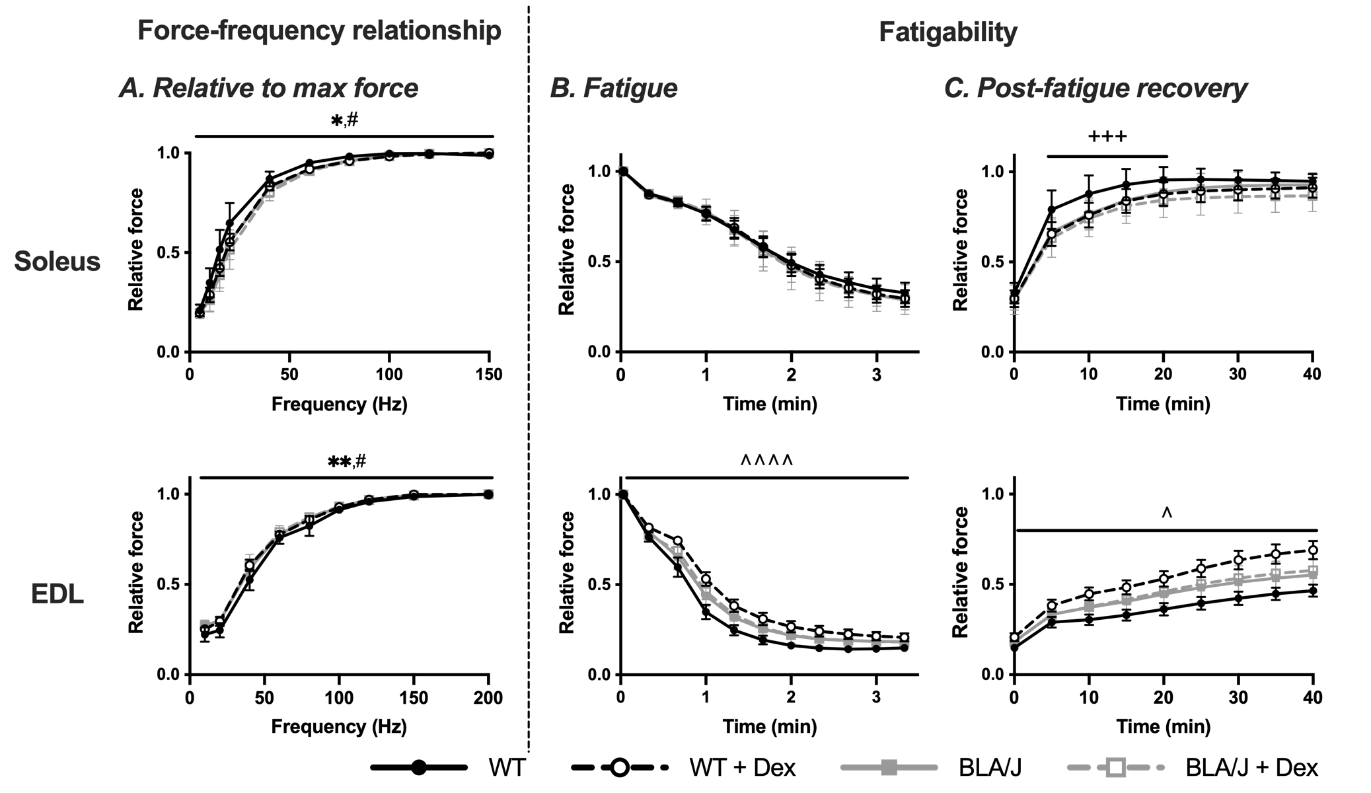


**Supplementary Fig. 11: Force and fatigue measurements for soleus and EDL muscles of WT and dysferlin-deficient BLA/J mice aged 10 months, without and with dexamethasone (Dex) treatment.** (A) Force-frequency relationship, normalised to maximum tetanic force, (B) fatigue and (C) post-fatigue recovery normalised to tetanic force at t = 0. For the isometric fatigue protocol, muscles were stimulated once every 2 s for 100 stimulations, with 800 ms of pulses at 60 Hz for the soleus and 500 ms of pulses at 70 Hz for the EDL. Post-fatigue recovery was recorded at 5-min intervals for 40 mins. Data were analysed by 3-way ANOVA: ***Soleus:*** * BLA/J (± Dex) lower than WT (± Dex) (*p* < 0.05, strain effect); # Dex-treated lower than untreated (*p* < 0.05, treatment effect); +++ WT + Dex lower than WT, BLA/J unaffected (*p* < 0.001, strain/treatment/time interaction effect). ***EDL:*** ** BLA/J (± Dex) higher than WT (± Dex) (*p* < 0.01, strain effect); # Dex-treated higher than untreated (*p* < 0.05, treatment effect); ^, ^^^^ WT + Dex higher than WT, BLA/J unaffected (*p* < 0.05, 0.0001, respectively, strain/treatment interaction effect). Data are presented as mean ± SD (*n* = 8–9). *Note: the data for untreated WT and BLA/J muscles has been published previously (55).*

**
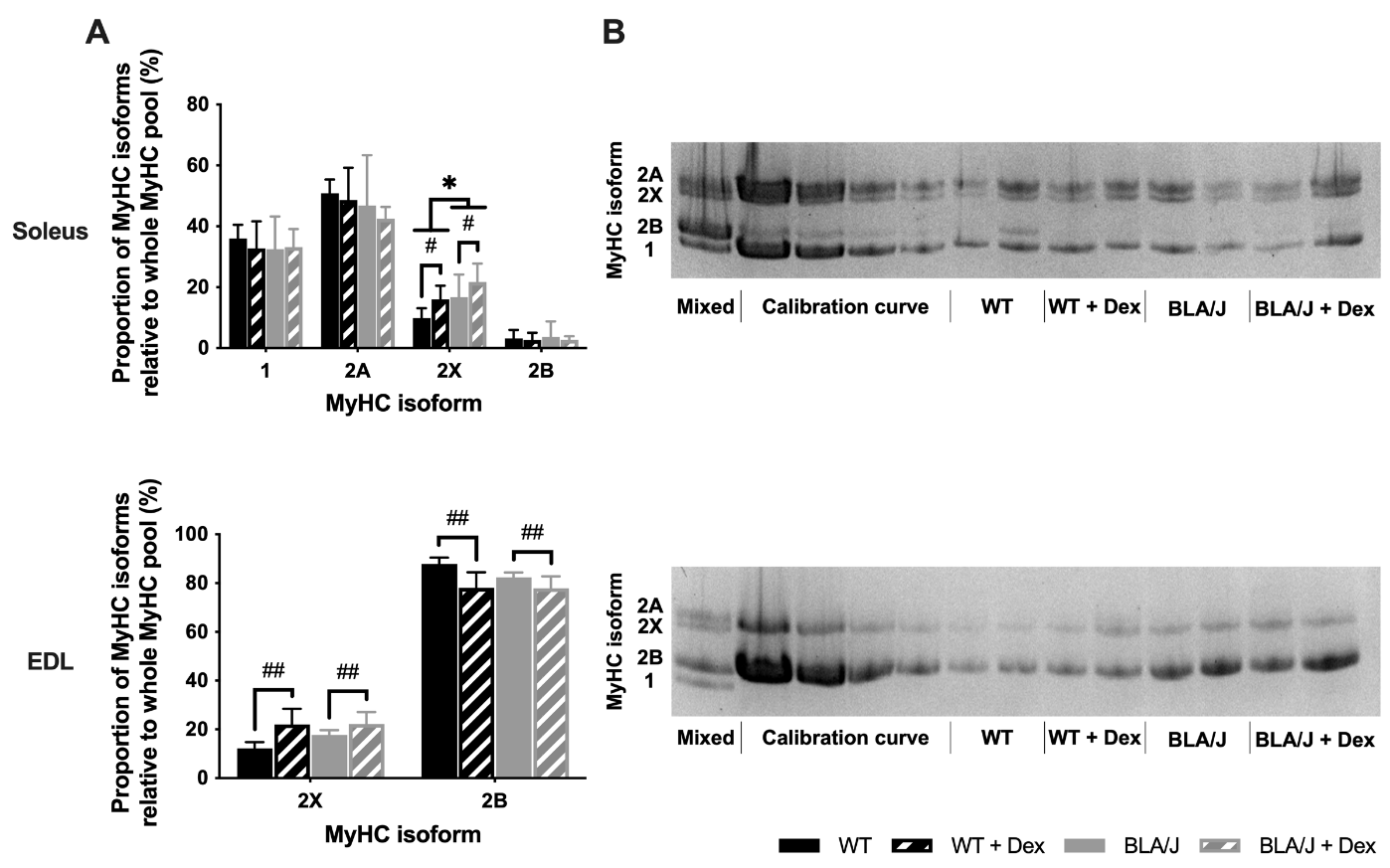
**

**Supplementary Fig. 12: Skeletal myofibre myosin heavy chain (MyHC) protein composition of soleus and EDL muscles of WT and dysferlin-deficient BLA/J mice aged 10 months, without and with dexamethasone (Dex) treatment.** (A) Percentage of different MyHC in soleus and EDL muscles with (B) representative MyHC gels loaded with a pooled sample (Mixed) used to identify all isoforms and a mixed sample to generate the Calibration curve. Data were analysed by two-way ANOVA: * BLA/J (± Dex) vs WT (± Dex) (*p* < 0.05, strain effect). #, ## Dex-treated vs untreated (*p* < 0.05, 0.01, respectively, treatment effect). Data are presented as mean ± SD (*n* = 6). *Note: the data for untreated WT and BLA/J muscles has been published previously (55).*

Biochemical protein analyses: soleus and EDL

**
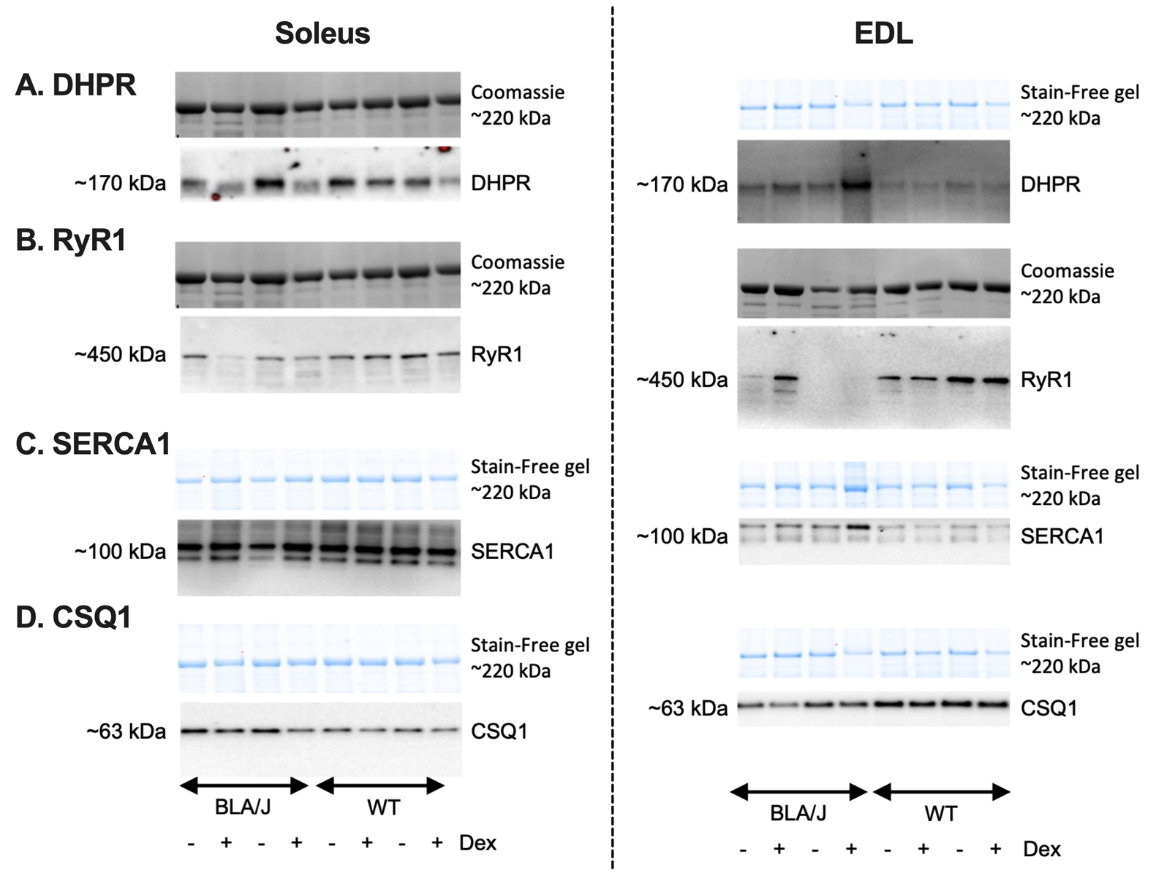
**

**Supplementary Fig. 13: Representative western blots for Ca^2+^ handling-associated proteins in soleus and EDL muscles of WT and dysferlin-deficient BLA/J mice aged 10 months, without (-) and with (+) dexamethasone (Dex) treatment.** (A) Dihydropyridine receptor (DHPR; at 170 kDa); (B) Ryanodine receptor 1 (RyR1; at 450 kDa); (C) Sarco/endoplasmic reticulum Ca^2+^-ATPase (SERCA1; at 100 kDa); (D) Calsequestrin 1 (CSQ1; at 63 kDa). Stain-Free and Coomassie gels show a region around 220 kDa, and the prominent band is likely myosin.

**
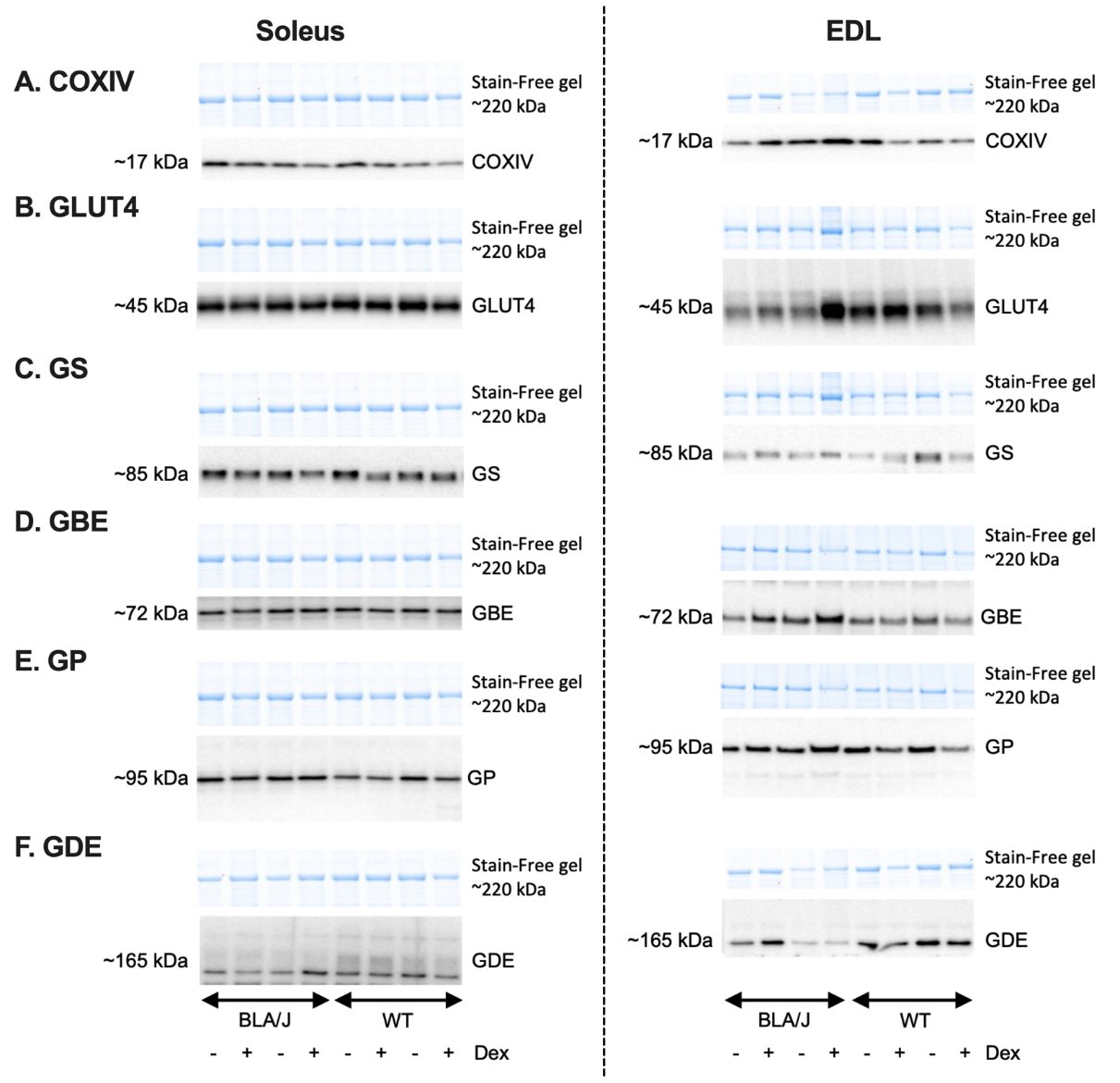
**

**Supplementary Fig. 14: Representative western blots for metabolism-associated proteins in soleus and EDL muscles of WT and dysferlin-deficient BLA/J mice aged 10 months, without (-) and with (+) dexamethasone (Dex) treatment.** Whole muscle homogenates analysed by western blot. (A) Cytochrome c oxidase subunit (COXIV; at 17 kDa); (B) Glucose transporter type 4 (GLUT4; at 45 kDa); (C) Glycogen synthase (GS; at 85 kDa); (D) Glycogen branching enzyme (GBE; at 72 kDa); (E) Glycogen phosphorylase (GP; at 95 kDa); (F) Glycogen debranching enzyme (GDE; at 165 kDa). Stain-Free gels show a region around 220 kDa, and the prominent band is likely myosin.

**Supplementary Table 2: Summary of levels of proteins associated with Ca^2+^ handling and glucose/glycogen metabolism in soleus and EDL muscles WT and dysferlin-deficient BLA/J male mice aged 10 months, without and with dexamethasone (Dex) treatment.** Whole muscle homogenates analysed by western blot. The amount of target protein in the given sample was normalised to the total protein in that lane and then expressed relative to the average of the soleus WT samples on a given gel (a.u.). Each data value is the mean of 3–4 independent measurements of a given muscle homogenate. For the RyR1 protein in the untreated BLA/J EDL muscles, this protein was below detection levels for 5 of the 6 muscles analysed, and therefore only a single value is shown. Data were analysed by two-way ANOVA: * BLA/J (± Dex) vs WT (± Dex) (*p* < 0.05, strain effect). Data are presented as mean ± SD (*n* = 4–6).

|  | **SOLEUS** | | | | | **EDL** | | | | |
| --- | --- | --- | --- | --- | --- | --- | --- | --- | --- | --- |
| **DETECTED PROTEINS** | **WT**  **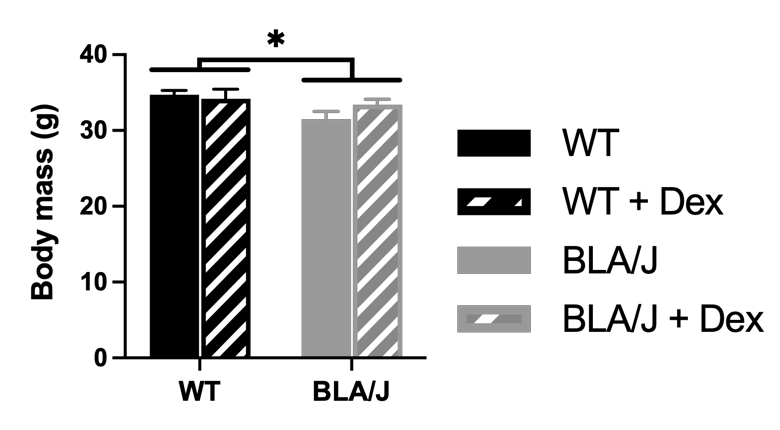** | **WT + Dex**  **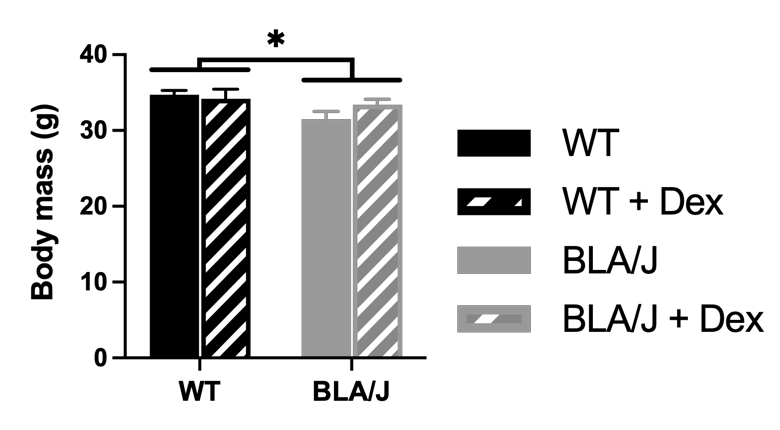** | **BLA/J**  **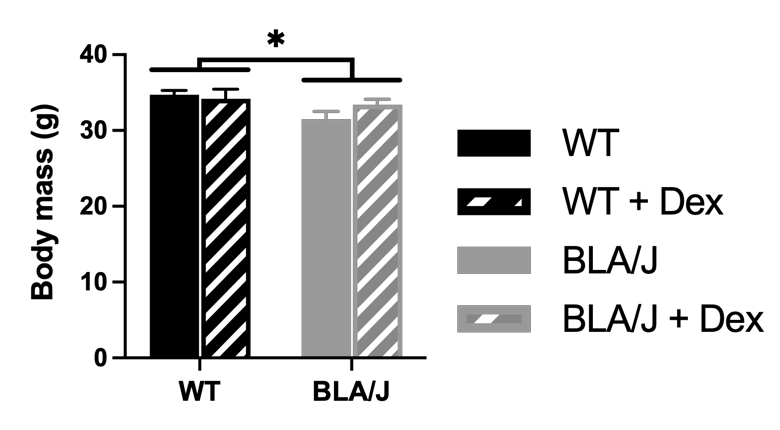** | **BLA/J + Dex**  **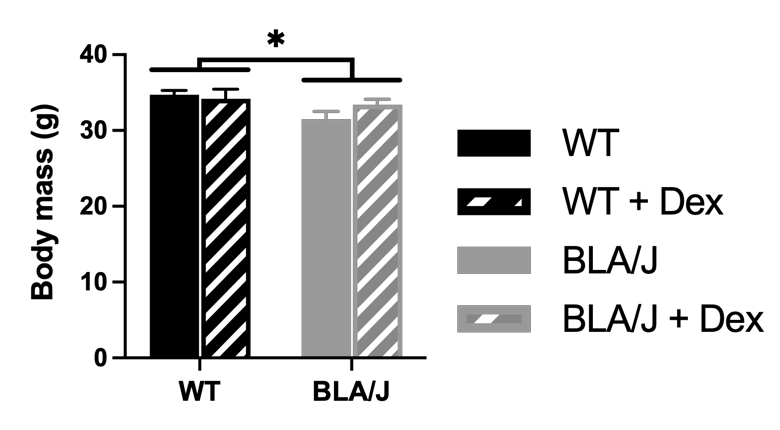** |  | **WT**  **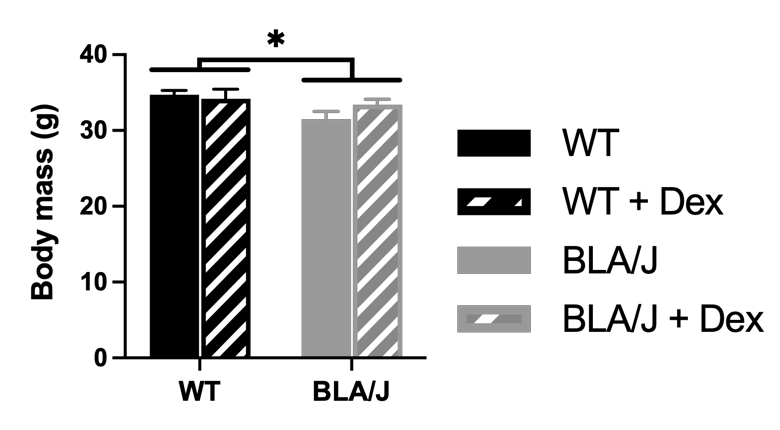** | **WT + Dex**  **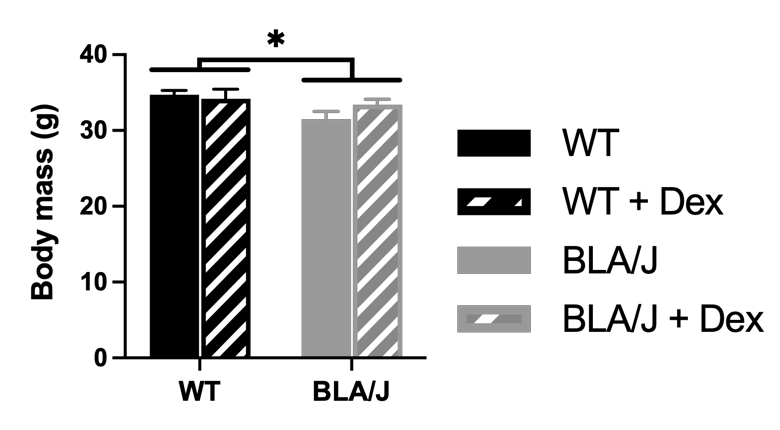** | **BLA/J**  **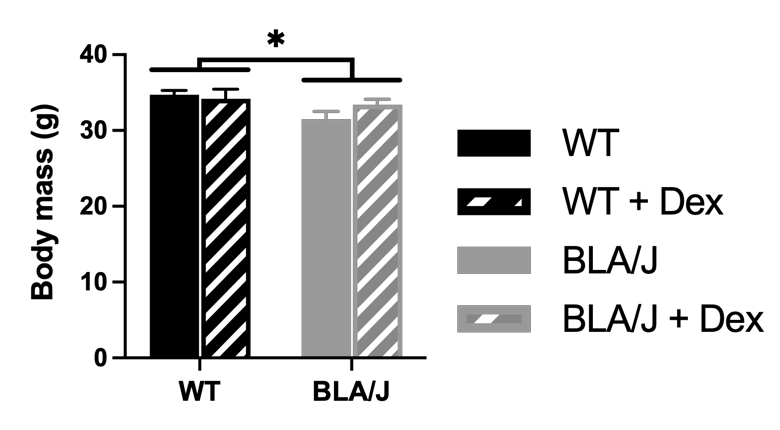** | **BLA/J + Dex**  **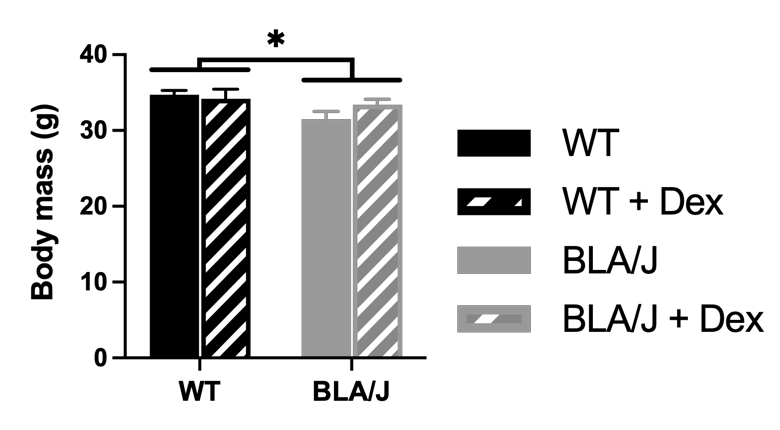** |  |
| **Ca^2+^ handling-associated** |  |  |  |  |  |  |  |  |  |  |
| *EC coupling* |  |  |  |  |  |  |  |  |  |  |
| DHPR | 1.02 ± 0.27 | 1.02 ± 0.27 | 1.09 ± 0.17 | 1.1 ± 0.28 |  | 0.88 ± 0.25 | 1 ± 0.26 | 5.27 ± 1 | 6.92 ± 0.59 | * |
| RyR1 | 1 ± 0.26 | 0.96 ± 0.25 | 0.81 ± 0.13 | 0.58 ± 0.11 | * | 1.05 ± 0.33 | 1 ± 0.3 | 0.18 ± 0.07 | 0.31 ± 0.12 | * |
| *Ca^2+^reuptake* |  |  |  |  |  |  |  |  |  |  |
| SERCA1 | 1 ± 0.27 | 1.04 ± 0.3 | 0.78 ± 0.1 | 0.87 ± 0.12 |  | 0.81 ± 0.24 | 1.01 ± 0.27 | 1.34 ± 0.26 | 1.26 ± 0.14 | * |
| *Ca^2+^ buffering* |  |  |  |  |  |  |  |  |  |  |
| CSQ1 | 1 ± 0.26 | 0.92 ± 0.24 | 1.43 ± 0.09 | 1.21 ± 0.08 | * | 1.12 ± 0.3 | 1 ± 0.26 | 0.96 ± 0.2 | 0.77 ± 0.09 | * |
| **Metabolism-associated** |  |  |  |  |  |  |  |  |  |  |
| *Aerobic enzymes* |  |  |  |  |  |  |  |  |  |  |
| COXIV | 1 ± 0.27 | 0.94 ± 0.26 | 1.23 ± 0.13 | 1.15 ± 0.11 |  | 0.9 ± 0.25 | 1 ± 0.26 | 1.16 ± 0.23 | 1.2 ± 0.11 | * |
| *Glucose uptake* |  |  |  |  |  |  |  |  |  |  |
| GLUT4 | 1 ± 0.26 | 0.87 ± 0.23 | 0.84 ± 0.1 | 0.91 ± 0.05 |  | 1.1 ± 0.29 | 1 ± 0.26 | 0.89 ± 0.17 | 0.87 ± 0.09 | * |
| *Glycogen storage* |  |  |  |  |  |  |  |  |  |  |
| GS | 1 ± 0.26 | 0.97 ± 0.27 | 0.82 ± 0.04 | 0.79 ± 0.03 | * | 1.19 ± 0.32 | 1 ± 0.27 | 0.97 ± 0.24 | 1.03 ± 0.11 |  |
| GBE | 1 ± 0.26 | 0.98 ± 0.26 | 0.98 ± 0.17 | 0.92 ± 0.12 |  | 1.14 ± 0.3 | 1 ± 0.26 | 0.78 ± 0.21 | 0.74 ± 0.12 | * |
| *Glycogen utilisation* |  |  |  |  |  |  |  |  |  |  |
| GP | 1 ± 0.26 | 0.97 ± 0.26 | 1.14 ± 0.1 | 1.18 ± 0.06 | * | 0.79 ± 0.24 | 1 ± 0.26 | 0.76 ± 0.19 | 0.96 ± 0.17 |  |
| GDE | 1 ± 0.29 | 0.9 ± 0.27 | 1.11 ± 0.25 | 0.89 ± 0.16 |  | 1.03 ± 0.29 | 1 ± 0.3 | 0.43 ± 0.12 | 1.53 ± 0.45 |  |
| **Abbreviations:**  ***Ca^2+^ handling-associated proteins:*** DHPR: dihydropyridine receptor; RyR1: ryanodine receptor 1; CSQ1: calsequestrin 1; SERCA1: sarco/endoplasmic reticulum Ca^2+^-ATPase.  ***Metabolism-associated proteins:*** cytochrome c oxidase subunit; GLUT4: glucose transporter type 4; COXIV GS: glycogen synthase; GBE: glycogen branching enzyme; GP: glycogen phosphorylase; GDE: glycogen debranching enzyme. | | | | | | | | | | |
